# Antibacterial Properties of Polyphenols: Characterization and QSAR (Quantitative Structure–Activity Relationship) Models

**DOI:** 10.3389/fmicb.2019.00829

**Published:** 2019-04-18

**Authors:** Lynda Bouarab-Chibane, Valérian Forquet, Pierre Lantéri, Yohann Clément, Lucie Léonard-Akkari, Nadia Oulahal, Pascal Degraeve, Claire Bordes

**Affiliations:** ^1^Univ Lyon, Université Claude Bernard Lyon 1, ISARA Lyon, BioDyMIA (Bioingénierie et Dynamique Microbienne aux Interfaces Alimentaires), Equipe Mixte d'Accueil n°3733, IUT Lyon 1, Technopole Alimentec, Bourg-en-Bresse, France; ^2^Univ Lyon, Université Claude Bernard Lyon 1, CNRS, ISA (Institut des Sciences Analytiques), UMR CNRS n°5280, Villeurbanne, France; ^3^Univ Lyon, Université Claude Bernard Lyon 1, LAGEPP (Laboratoire d'Automatique, de Génie des Procédés et de Génie Pharmaceutique), UMR CNRS n°5007, Villeurbanne, France

**Keywords:** polyphenols, antibacterial activity, quantitative structure activity relationships (QSAR), foodborne pathogenic bacteria, food-spoiling bacteria, surface properties of bacteria

## Abstract

Besides their established antioxidant activity, many phenolic compounds may exhibit significant antibacterial activity. Here, the effect of a large dataset of 35 polyphenols on the growth of 6 foodborne pathogenic or food-spoiling bacterial strains, three Gram-positive ones (*Staphylococcus aureu*s, *Bacillus subtilis*, and *Listeria monocytogenes*) and three Gram-negative ones (*Escherichia coli, Pseudomonas aeruginosa*, and *Salmonella* Enteritidis), have been characterized. As expected, the effects of phenolic compounds were highly heterogeneous ranging from bacterial growth stimulation to antibacterial activity and depended on bacterial strains. The effect on bacterial growth of each of the polyphenols was expressed as relative Bacterial Load Difference (BLD) between a culture with and without (control) polyphenols at a 1 g L^−1^ concentration after 24 h incubation at 37°C. Reliable Quantitative Structure-Activity Relationship (QSAR) models were developed (regardless of polyphenol class or the mechanism of action involved) to predict BLD for *E. coli, S*. Enteritidis, *S. aureu*s, and *B. subtilis*, unlike for *L. monocytogenes* and *P. aeruginosa*. *L. monocytogenes* was generally sensitive to polyphenols whereas *P. aeruginosa* was not. No satisfactory models predicting the BLD of *P. aeruginosa* and *L. monocytogenes* were obtained due to their specific and quite constant behavior toward polyphenols. The main descriptors involved in reliable QSAR models were the lipophilicity and the electronic and charge properties of the polyphenols. The models developed for the two Gram-negative bacteria (*E. coli, S*. Enteritidis) were comparable suggesting similar mechanisms of toxic action. This was not clearly observed for the two Gram-positive bacteria (*S. aureu*s and *B. subtilis*). Interestingly, a preliminary evaluation by Microbial Adhesion To Solvents (MATS) measurements of surface properties of the two Gram-negative bacteria for which QSAR models were based on similar physico-chemical descriptors, revealed that MATS results were also quite similar. Moreover, the MATS results of the two Gram-positive bacterial strains *S. aureus* and *B. subtilis* for which QSARs were not based on similar physico-chemical descriptors also strongly differed. These observations suggest that the antibacterial activity of most of polyphenols likely depends on interactions between polyphenols and bacterial cells surface, although the surface properties of the bacterial strains should be further investigated with other techniques than MATS.

## Introduction

Besides their established antioxidant activity, many phenolic compounds may exhibit significant antibacterial activity. Since many plant extracts are rich in phenolic compounds, this is of particular interest for the development of natural alternatives to synthetic preservatives in food (Bouarab-Chibane et al., 2019) and cosmetic applications (Kocevar Glavac and Lunder, 2018).

The mechanisms of antibacterial action of phenolic compounds are not yet fully deciphered but these compounds are known to involve many sites of action at the cellular level (Sikkema et al., 1995). Several authors explained this activity by the modification in permeability of cell membranes, the changes in various intracellular functions induced by hydrogen binding of the phenolic compounds to enzymes or by the modification of the cell wall rigidity with integrity losses due to different interactions with the cell membrane (Ikigai et al., 1993; Stapleton et al., 2004; Taguri et al., 2006; Cushnie and Lamb, 2011). Thus, the elevation of the lipophilic character of phenolic compounds enhances their antimicrobial activity by favoring their interaction with the cell membrane (Sikkema et al., 1995). This may induce irreversible damages of the cytoplasmic membrane and coagulation of the cell content that can even lead to the inhibition of intracellular enzymes. For example, condensed phenylpropanoids—tannins may induce damages at the cell membrane and even inactivate the metabolism by binding to enzymes (Ya et al., 1988; Chung et al., 1998) while phenolic acids have been shown to disrupt membrane integrity, as they cause consequent leakage of essential intracellular constituents (Borges et al., 2013). Flavonoids may link to soluble proteins located outside the cells and with bacteria cell walls thus promoting the formation of complexes (Tsuchiya et al., 1996; Cushnie and Lamb, 2011). Flavonoids also may act through inhibiting both energy metabolism and DNA synthesis thus affecting protein and RNA syntheses (Haraguchi et al., 1998). In the case of Gram-positive bacteria, intracellular pH modification as well as interference with the energy (ATP) generating system were reported (Djilani and Dicko, 2012).

Despite the complexity of the mechanisms of action involved, different authors have investigated the antibacterial activity of phenolic compounds by Quantitative Structure-Activity Relationship (QSAR) studies. Most of these studies concern essential oils (i.e., volatile phenolic compounds due to their low molecular weight). These studies have shown the importance of the contribution of the octanol-water partition coefficient (Log P) in relation with the hydrophobic and amphiphilic character of the molecule (Beltrame et al., 1988; Sierra-Alvarez and Lettinga, 1991; Oyedemi et al., 2009), the role of the number and the position of OH groups (Griffin et al., 2005), the role of size and type of alkyl groups (Pelczar et al., 1988; Dorman and Deans, 2000), and the contribution of the presence of acetate groups (Dorman and Deans, 2000) and aldehydes (Kurita et al., 1981; Moleyar and Narasimham, 1986) in the antibacterial efficacy of phenolic compounds. These factors influencing the antibacterial activity of essential oil chemotypes are summarized and analyzed in the two reviews of Radulovic et al. (2013) and Gyawali and Ibrahim (2014). The existing literature concerning QSARs for the prediction of the antibacterial activity of phenolic compounds of slightly higher molecular weight such as phenolic acids, flavonoids, stilbenes, coumarins and quinones still remains limited (Cushnie and Lamb, 2011; Daglia, 2012; Duggirala et al., 2014; Li et al., 2014; Lumbiny et al., 2014; Upadhyay et al., 2014; Fang et al., 2016). Although a QSAR study has not been conducted regarding the antibacterial effect of polyphenols (Larif et al., 2015), descriptors related to the number of hydroxyl functions, electronic effects and lipophilicity are the most common in QSAR models involving polyphenols.

In this context, the aim of this study was to determine the antimicrobial effect of 35 polyphenols belonging to different classes (cinnamic or benzoic acids, flavonoids, stilbenes, coumarins, naphtoquinones) against six foodborne pathogenic or food-spoiling bacterial strains: three Gram-positive ones, *S. aureu*s CNRZ3, *B. subtilis* ATCC6633, and *L. monocytogenes* ATCC19115, and three Gram-negative ones, *E. coli* ATCC25922, *P. aeruginosa* ATCC27853, and *S*. Enteritidis E0220. The percentage reduction in optical density at 420–580 nm after 24 h incubation at 37°C in the presence of 1 g L^−1^ of each polyphenol, also called Bacterial Load Difference (BLD) (%), was the parameter retained to express the antimicrobial activity. QSAR models were then developed to try to predict this property and thus highlight the structural characteristics of the polyphenols that should condition their antimicrobial activity. Considering BLD in the presence of a 1 g L^−1^ concentration of each phenolic rather than Minimal Inhibitory or Bactericidal (MIC or MBC) as a descriptor of the effect of each phenolic on bacterial growth allowed to build a QSAR model predicting the low antibacterial activity, the absence of antibacterial activity as well as the bacterial growth promoting effect sometimes observed with some phenolics. A 1 g L^−1^ concentration of polyphenol was chosen in order to identify polyphenols with a significant *in vitro* antibacterial activity at this concentration: indeed, it was considered that polyphenols which would be active at higher concentrations in Mueller-Hinton broth would have no practical interest for *in situ* applications such as addition into food matrices, since far higher concentrations than *in vitro* are generally necessary for perishable foods preservation (Miceli et al., 2014). In order to check whether the most influential physico-chemical parameters conditioning the antibacterial activity of polyphenols in QSAR models were correlated with the physico-chemical surface properties of bacterial cells surface, a very preliminary study of their surface properties was performed by measuring microbial adhesion to 4 solvents for each bacterial strain.

## Materials and Methods

### Materials

The dataset was made of 35 polyphenols belonging to different phenolic classes: 3 stilbenes (rhapontin, resveratrol, pinosylvin), 8 cinnamic (caffeic acid, caffeic acid 1,1-dimethylallyl ester, chicoric acid, cinnamyl-3,4-dihydroxy-α-cyanocinnamate, 2,4-dihydroxycinnamic acid, ethyl 3,4-dihydroxycinnamate, chlorogenic acid, CU-CPT22 acid) and 6 benzoic (butyl gallate, ethyl 3,5-dihydroxybenzoate, 3,4-dihydroxy-benzoic acid methyl ester, 2,4-dihydroxy-3,6-dimethylbenzoic acid, isopropyl 3,4,5-trihydroxybenzoate, methyl 3,5 dihydroxybenzoate) acids, 11 flavonoids (cardamonin, dihydromyricetin, diosmin, epigallocatechin gallate, myricetin, myricitrin, quercetin 3-β-D-glucoside, rutin, silibinin, taxifolin, wedelolactone), 5 coumarins (baicalein, 3′,5′-dihydroxyflavone, 5,7-dihydroxy-4-phenylcoumarin, 5,7-dihydroxy-4-propylcoumarin, 5,7-dihydroxy-4-methylcoumarin) and 2 naphtoquinones (5,8-dihydroxy-1,4-naphthoquinone, 2,3-dichloro-5,8-dihydroxy-1,4-naphthoquinone) (Table 1). For sake of clarity in Tables and Figures, polyphenols were described and ranked by their Inchikey (http://inchi.info/) shortened to the first three characters. Polyphenols, a 2.5% (w/w) nisin preparation, ciprofloxacin, dimethyl sulfoxide (DMSO) (PubChem CID: 679) (purity = 99.5%), chloroform, ethyl acetate, hexane, and hexadecane were purchased from Sigma Aldrich (St. Quentin Fallavier, France). Mueller Hinton Broth (MHB) was provided by Grosseron (Couëron, France).

**Table 1:** Structure and antibacterial properties of the 35 polyphenols studied.

**Shortened InChikey ID**	**Polyphenol**	**Chemical structure**		**Gram-negative bacteria**							**Gram-positive bacteria**						
				***E. coli*** **ATCC25922**		***S*****. Enteritidis E0220**		***P. aeruginosa*** **ATCC27853**		***S. aureus*** **CNRZ3**		***B. subtilis*** **ATCC6633**		***L. monocytogenes*** **ATCC19115**	
			**Molecular weight (g.mol^**−1**^)**	**BLD% (average)**	SD^*^	**BLD% (average)**	SD^*^	**BLD% (average)**	SD^*^	**BLD% (average)**	SD^*^	**BLD% (average)**	SD^*^	**BLD% (average)**	SD^*^
APH	Ethyl-3,5-dihydroxy-benzoate	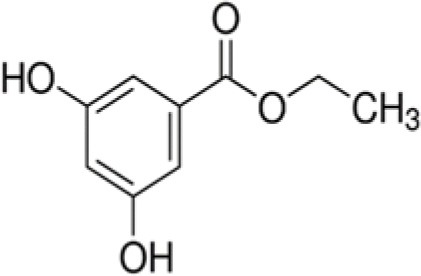	182	77.3	2.2	85.4	3.5	26.8	1.9	29.4	1.2	82.2	5.9	17.5	11.7
CUF	3,4-dihydroxy-benzoic acid methyl ester	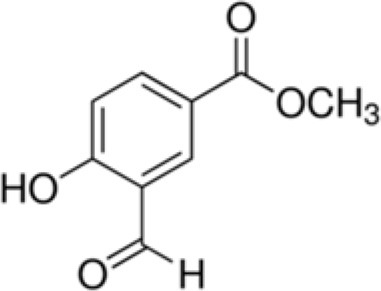	168	74.0	4.2	82.4	5.3	7.4	5.9	18.2	7.3	69.3	4.2	58.6	10.5
CWV	Chlorogenic acid	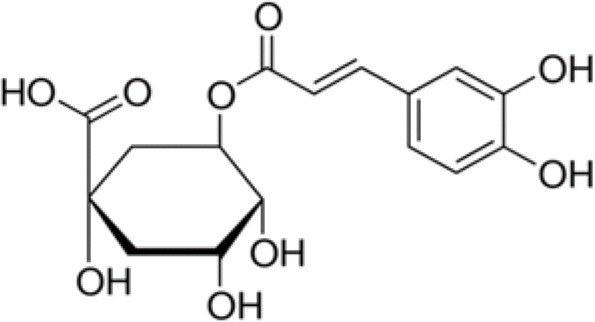	354	−4.2	1.1	5.0	3.8	5.6	4.7	10.6	4.8	−21.4	5.9	43.4	22.3
CXQ	Taxifolin	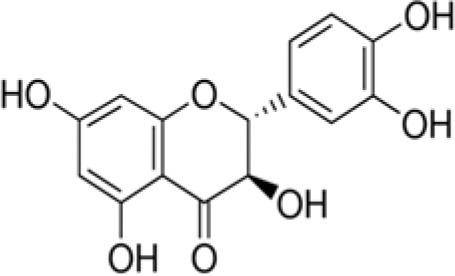	304	18.1	3.1	20.8	4.6	10.4	1.6	87.7	4.8	24.9	11.7	69.4	8.1
DCY	Myricitrin dihydrate	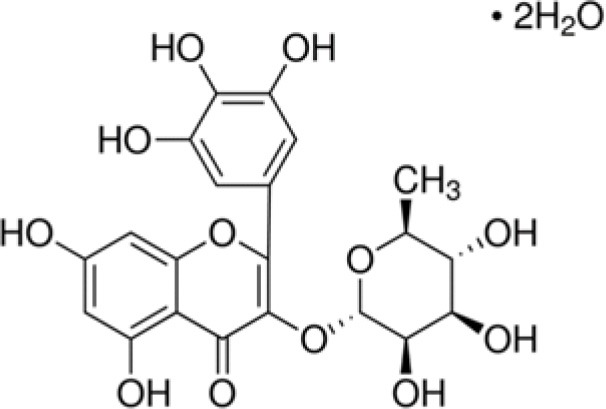	500	0.5	9.2	−4.3	0.4	−3.5	6.5	−0.3	0.5	−67.8	7.3	58.5	2.5
FXN	Baicalein	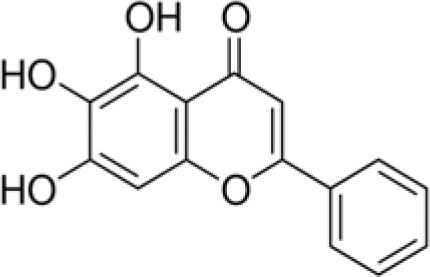	270	22.9	17.6	27.2	14.5	25.7	14.8	100.0	–	36.2	2.8	52.7	–
GKA	Rhapontin	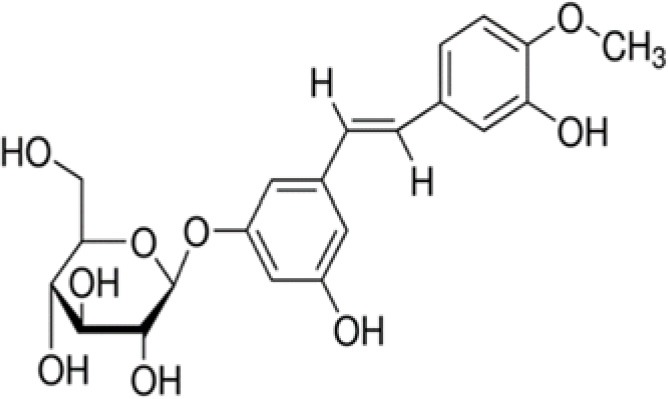	420	−5.5	5.2	−15.0	3.0	10.8	1.9	−9.7	7.4	−5.8	20.7	16.7	8.4
GZS	Diosmin	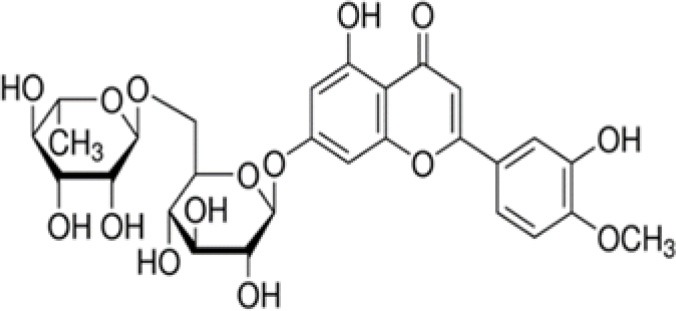	609	−28.6	5.6	56.4	–	−7.6	5.1	42.5	7.3	−13.2	3.8	44.2	14.0
HDP	5,7-dihydroxy-4-propylcoumarin	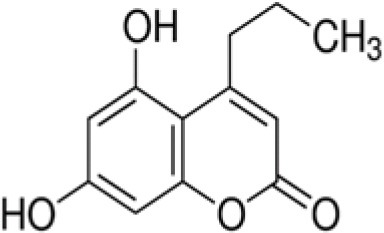	220	−1.8	9.8	16.8	13.0	3.5	9.2	44.8	8.4	21.2	7.0	46.9	15.2
HGE	2,4-dihydroxy-cinnamic acid	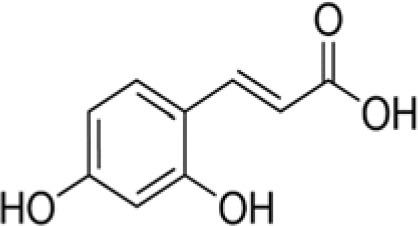	180	−7.8	4.0	27.8	7.8	1.7	3.5	15.6	8.2	−3.6	2.5	38.3	14.4
HUQ	5,7-dihydroxy-4-phenylcoumarin	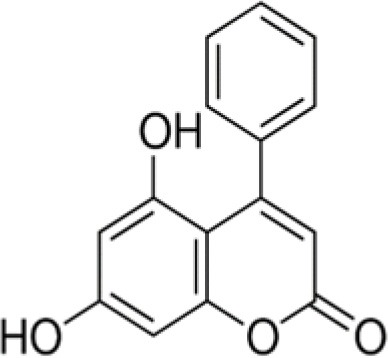	254	−26.1	4.0	38.3	8.9	19.3	1.3	89.9	7.9	51.5	4.9	93.5	8.0
IKG	Rutin hydrate	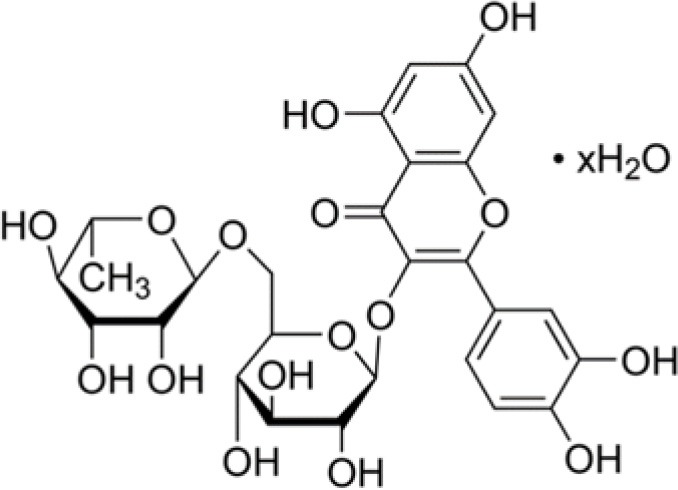	611	68.0	14.0	36.8	21.5	60.7	8.9	32.7	10.1	100.0	–	23.3	9.0
IKM	Myricetin	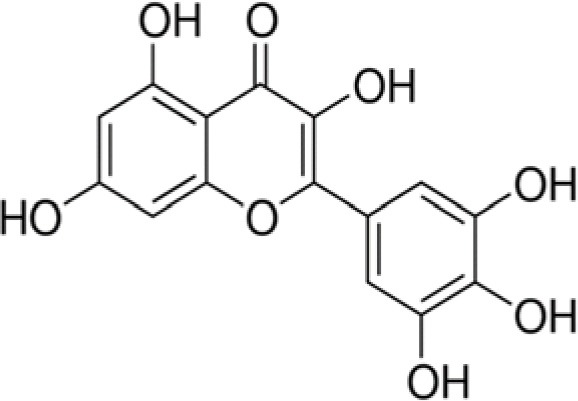	318	46.2	17.1	41.0	18.5	28.3	19.2	54.9	20.1	21.5	28.7	36.5	20.8
KJX	Dihydromyricetin	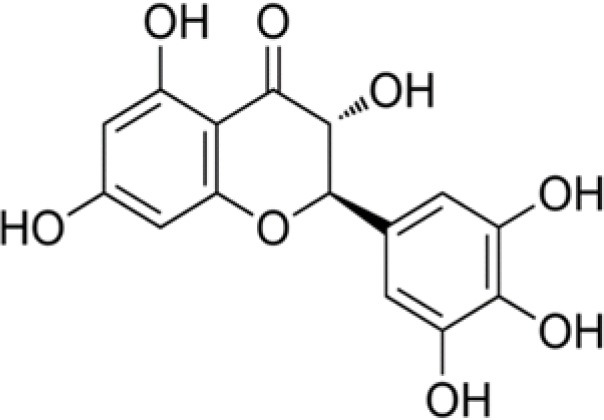	320	1.1	10.0	18.8	3.4	4.8	10.7	39.1	8.3	14.6	9.1	43.7	3.7
LUK	Resveratrol	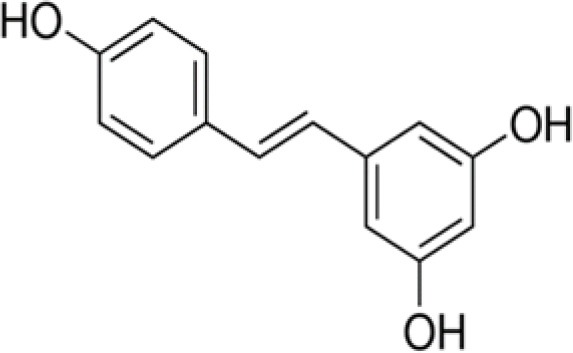	228	100.0	–	100.0	–	60.2	9.1	100.0	–	75.2	16.9	100.0	–
MCC	3',5'-dihydroxyflavone	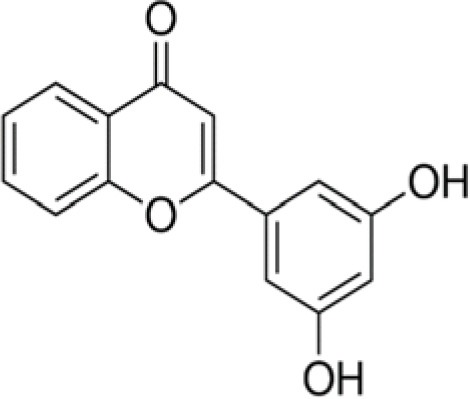	254	49.0	1.4	79.2	0.1	72.4	0.5	98.4	–	39.6	26.3	100.0	–
NYS	Cardamonin	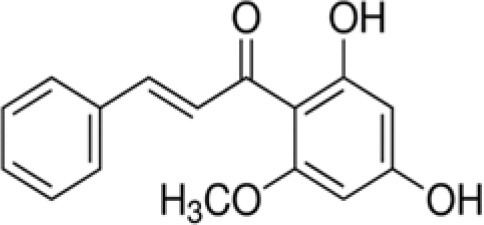	270	34.8	0.8	28.7	20.1	38.7	21.6	96.1	29.5	61.9	9.2	51.7	–
OVS	Quercetin 3-β-D-glucoside	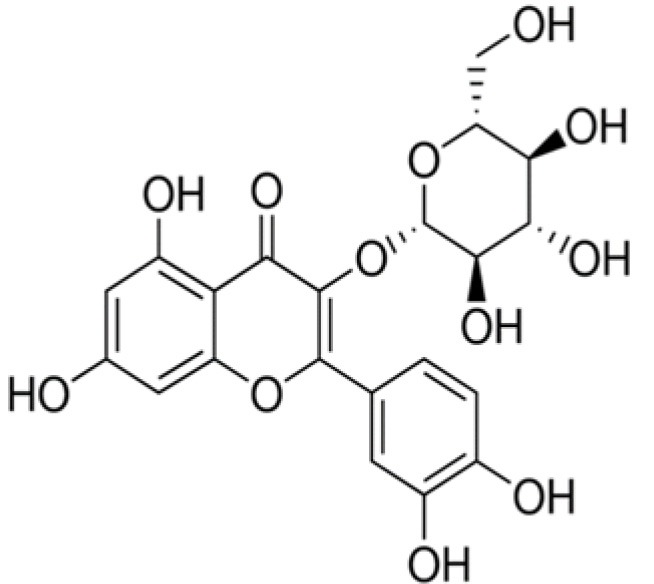	464	3.8	12.2	−12.5	7.4	16.2	1.7	50.8	15.9	17.2	9.6	75.1	6.2
QAI	Caffeic acid	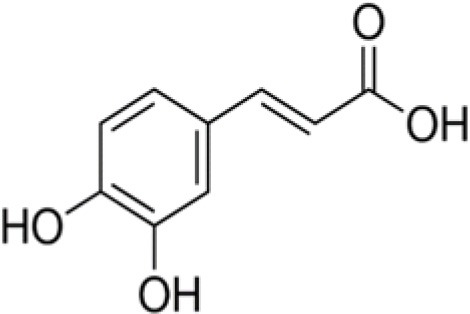	180	−22.2	4.9	68.6	0.9	81.8	12.9	40.2	13.0	22.5	10.1	35.2	4.5
QNV	5,7-dihydroxy-4-methylcoumarin	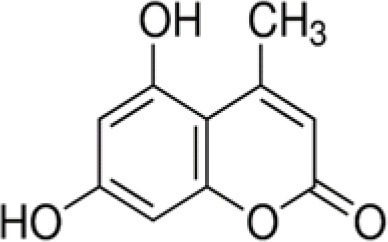	192	10.3	8.9	33.3	15.8	28.7	0.8	19.3	22.8	−2.5	5.3	97.9	2.1
RNV	Methyl-3,5-dihydroxy-benzoate	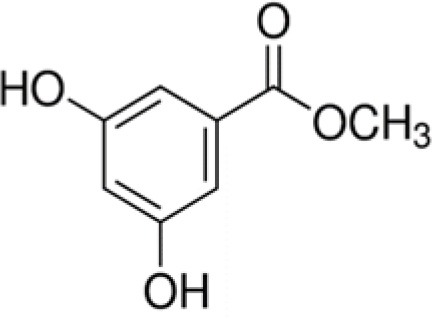	168	100.0	–	66.3	11.7	23.0	3.8	24.0	12.4	74.3	8.3	22.8	4.0
RQN	5,8-dihydroxy-1,4-naphthoquinone	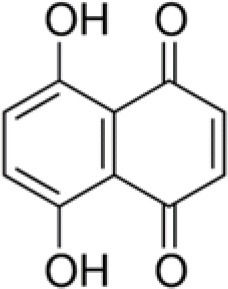	190	89.6	7.6	100.0	–	35.0	3.0	100.0	–	96.2	24.4	100.0	–
SEB	Silibinin	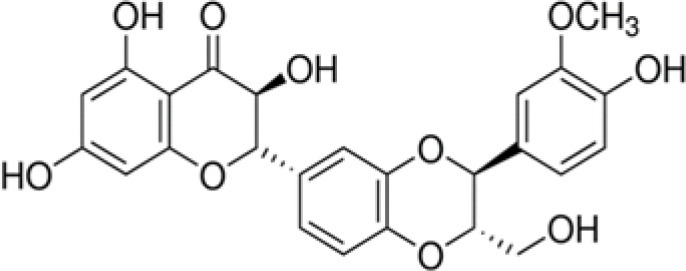	482	4.9	7.6	−19.1	3.4	12.6	3.1	8.2	6.6	17.0	1.4	9.4	8.9
TTY	Caffeic acid 1,1-dimethylallyl ester	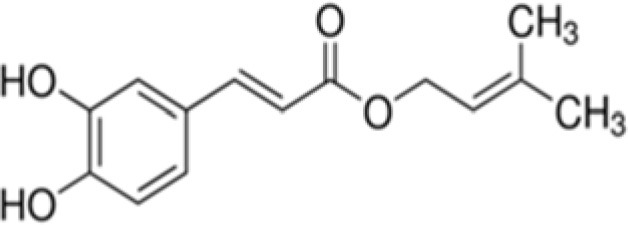	248	17.5	2.5	66.4	–	5.0	2.2	98.6	4.2	88.2	7.1	72.3	9.8
TXG	Isopropyl 3,4,5-trihydroxy-benzoate	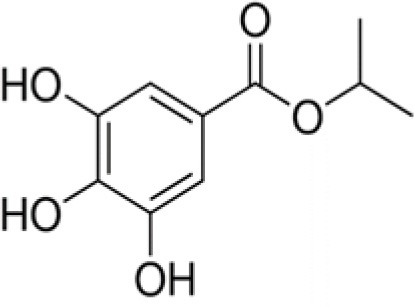	212	100.0	–	91.5	1.4	12.1	5.4	46.7	–	100.0	–	36.2	1.1
UBQ	CU-CPT22 acid	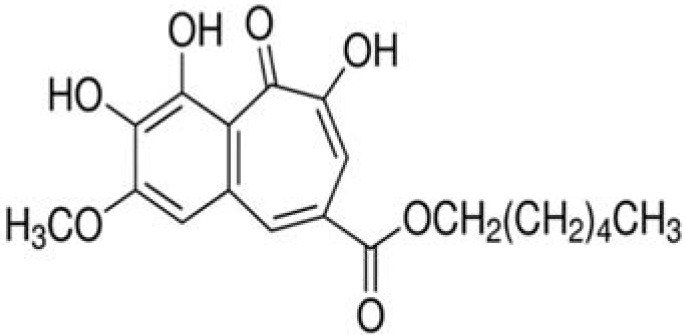	362	86.6	19.9	21.2	14.5	40.2	–	91.8	5.3	72.6	19.7	100.0	–
UVE	2,3-dichloro-5,8-dihydroxy-1,4-naphthoquinone	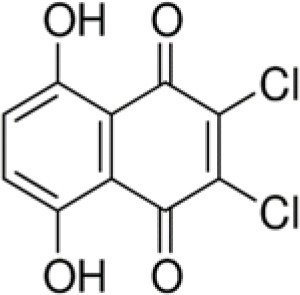	259	34.0	34.0	84.4	13.5	−5.3	22.1	80.6	9.9	67.3	12.1	100.0	–
VHN	2,4-dihydroxy-3,6-dimethylbenzoic acid	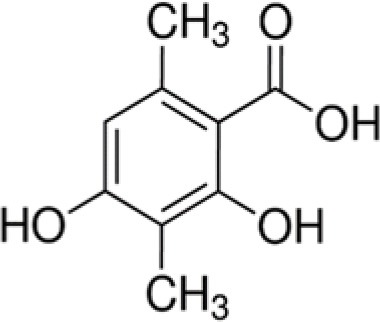	182	−29.1	2.5	58.2	3.4	−2.0	3.2	−2.2	10.4	−8.3	1.4	29.8	12.6
WDK	Ethyl-3,4-dihydroxy-cinnamate	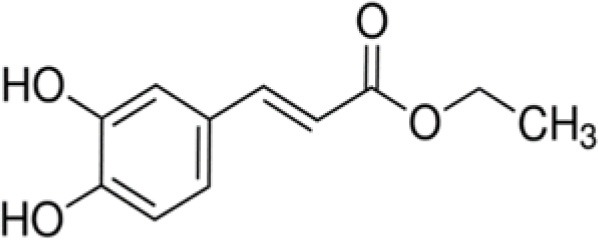	208	34.8	3.1	76.6	9.7	13.4	6.0	29.6	15.3	80.8	0.2	47.0	8.7
WMB	Epigallocatechin gallate	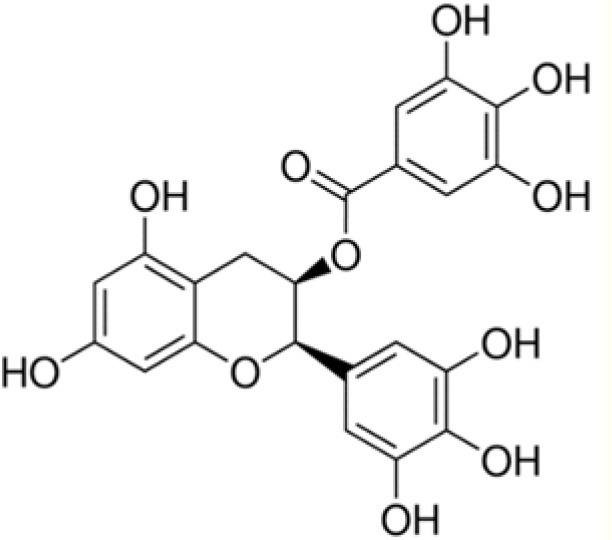	458	17.7	2.5	46.8	2.2	74.7	4.3	55.3	–	47.1	0.1	100.0	–
XGH	Cinnamyl-3,4-dihydroxy-α-cyanocinnamate	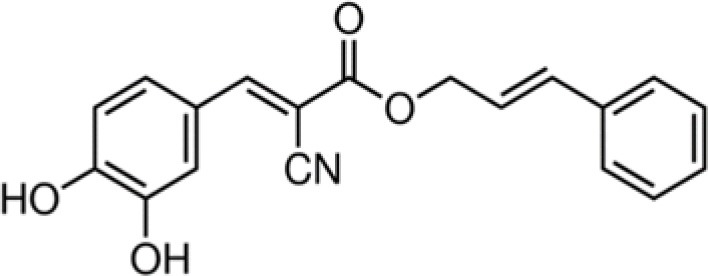	321	59.3	15.7	82.1	7.5	65.6	2.0	100.0	–	100.0	–	100.0	–
XOP	Butyl gallate	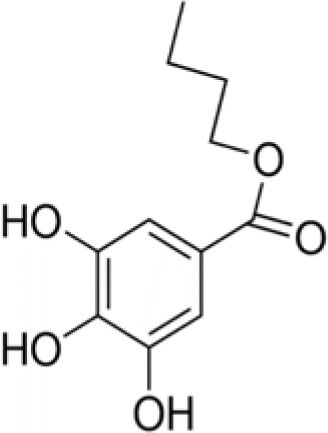	226	94.5	6.1	92.5	1.4	10.5	1.0	90.9	4.5	94.4	8.5	100.0	–
XQD	Wedelolactone	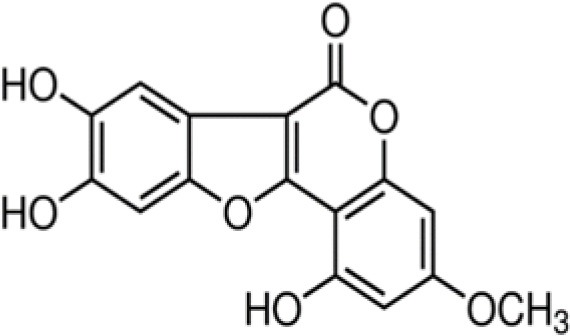	314	0.6	5.8	−23.6	1.3	−2.9	2.1	8.4	11.3	−15.2	2.1	21.6	–
YCV	Pinosylvin	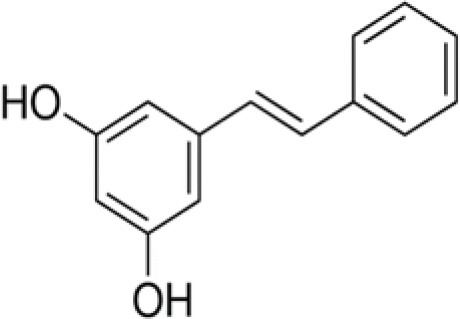	212	58.9	46.2	80.6	0.7	8.9	9.6	100.0	–	99.2	1.4	97.9	3.9
YDD	(-)-Chicoric acid	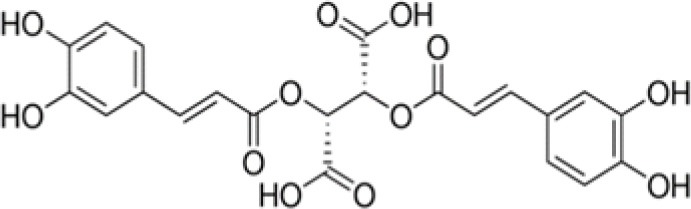	474	−17.1	6.1	35.2	3.6	12.0	7.5	24.7	2.5	7.6	3.1	53.5	6.5
		Estimation of the global SD (%)			12.8		8.2		8.6		8.9		11.5		9.8
		95% confidence interval (%)			±25		±17		±17		±17		±23		±20

* *SD is the standard deviation determined from the triplicates*.

### Determination of the Antibacterial Activity

The antibacterial activity of each phenolic compound was assessed by monitoring the cell growth of six bacterial strains (*S. aureus* CNRZ3, *B. subtilis* ATCC6633, *L. monocytogenes* ATCC19115, *E. coli* ATCC25922, *P. aeruginosa* ATCC27853, *S*. Enteritidis E0220), individually through the broth microdilution method, conducted as outlined in the National Committee for Clinical Laboratory Standards (2004). Briefly, 270 μL of MHB supplemented with phenolics (diluted in DMSO 1% (v/v) in distilled water) at a final 1 g L^−1^ concentration were mixed with 30 μL of bacterial inocula [5.0 10^6^ CFU (colony forming units) mL^−1^] in each well of the microplate and incubated at 37°C for 24 h in a Bioscreen C apparatus (Oy Growth Curves AB Ltd., Helsinki, Finland). Negative (MHB alone) and positive (MHB containing 2,000 IU mL^−1^ nisin for Gram-positive bacteria or ciprofloxacin 2 mg L^−1^ for Gram-negative bacteria) controls were also considered. The optical density of the culture was monitored every 15 min, in the 420–580 nm wavelength range (OD_420−580_).

The Bacterial Load Difference (BLD) observed in the presence of polyphenols was expressed by the percentage of reduction of OD_420−580_ after 24 h incubation at 37°C, calculated using the formula: BLD = Percent reduction of OD_420−580_ after 24 h of incubation = (1 – OD_420−580_ of test well/OD_420−580_ of corresponding control well) × 100.

### QSAR Development

#### Polyphenols Description

QSAR models require molecular descriptors. The 3D chemical structures of the polyphenols were initially built and optimized in gas phase using ADF (Amsterdam Density Functional) software (http://www.scm.com) and GAUSSIAN software (http://gaussian.com). These softwares are based on Density Functional Theory (DFT) which is the most efficient way to calculate accurate and reliable electronic properties of molecules. Descriptor calculations were performed with the PBE (Perdew et al., 1996) GGA (Generalized Gradient Approximation) exchange-correlation functional method and a TZP (triple zeta) basis set.

From the results of DFT calculations, 13 ADF descriptors and 9 GAUSSIAN descriptors were selected: the energy of the highest occupied molecular orbitals (HOMO and HOMO-1), the energy of the lowest unoccupied molecular orbitals (LUMO and LUMO-1), the polarizability, the maximal and minimal atomic Mulliken charges, the maximal and minimal atomic Hirshfeld charges, the hardness, the dipole moment, the molecule's ovality, the vertical ionization potential (v_I), the vertical electronic affinity (v_μ = –(I+A)/2), the softness (v_S = 1/η), the electrophilicity index (v_ω = μ2/2η), nucleophilicity (v_N-), nucleofugality (v_λ_N), hardness (water) (w_η = I–A), nucleofugality (water) (w_λ_N), and electrofugality (water) (w_λ_E).

In addition, the electrostatic potential (ESP) has been computed on the solvent accessible surface around the molecule. The surface around each atom is constructed from its van der Waals radius and that of the solvent (water) (Levet et al., 2013, 2016). The molecule surface was determined for given ESP values comprised between −0.2 eV and +0.1 eV with a step size of 0.01 eV. The parameter S corresponds to the percentage of cumulative surface between two ESP values. Regarding the polyphenols studied, five ESP zones were defined: S < −0.1 (eV), −0.1 <S < −0.05, −0.05 <S < 0, 0 <S < 0.05, and S> 0.05. Negative ESP values correspond to molecule regions having a Lewis base character, and positive ones to regions having a Lewis acid character. Polyphenols with a large positive and/or negative ESP surface are hydrophilic molecules. In contrast, molecules with a large surface area having ESP values centered around 0 exhibit a hydrophobic character.

To complete the description of the polyphenols, molecular properties and connectivity indices were calculated using Dragon software (http://chm.kode-solutions.net). Twenty descriptors were selected: saturation index (Ui), topological polar surface area (TPSA), Moriguchi octanol-water partition coeff. (MlogP), Ghose-Crippen octanol-water partition coeff. (AlogP), packing density index (PDI), different connectivity index (denoted X0A to X4aV), modified Randic index (XMOD), reciprocal distance sum Randic-like index (RDCHI), and LogD pH (5.5) which corresponds to the LogP partition coefficient obtained at pH 5.5.

Several physico-chemical properties (Log P, solubility) and geometrical descriptors such as H-donors, H-acceptors, ring counts, molecular shape, flexibility and complexity were also obtained from DataWarrior software (http://www.openmolecules.org).

By adding the molecular weight, the 35 polyphenols were characterized by a total of 59 descriptors.

#### Descriptor Selection

Multiple Linear Regression (MLR) QSARs were developed by using the Enhanced Replacement Method (ERM) (Mercader et al., 2008) with Matlab 7.9 Software (QSAR/QSPR search algorithms Toolbox; www.mathworks.fr/products/matlab/). MLR was chosen so as to obtain easily interpretable and applicable models.

Then, the optimum subset of descriptors to be included in the models was selected by the Kubinyi function (FIT) (Kubinyi, 1994). The FIT statistical parameter is closely related to the Fisher ratio F and is expressed as:

(1) $$\begin{array}{r} FIT=\frac{R^{2}\left(N-d-1\right)}{\left(N+d^{2}\right)\left(1-R^{2}\right)} \end{array}$$

where *R*^2^ is the determination coefficient, d the number of descriptors selected in the model, and N the number of molecules. The FIT parameter is preferred to F since the latter is too sensitive to changes in small d values and poorly sensitive to changes in large d values.

The optimal number of descriptors selected in the models corresponds to the maximum value of FIT in the plot FIT vs. d. The choice of the descriptors was confirmed by performing Student's *t*-test at a confidence level of 95%.

#### Model Validation

The determination coefficient *R*^2^ and the Root-Mean-Square Error (RMSE) were used as indicators for model quality. The predictive power and robustness of the models developed were assessed by internal and external validation techniques.

Five-fold Cross-Validation procedure was employed for model internal validation purpose. It consisted in splitting the dataset in 5 subsets of similar size. Four subsets were used as training set while the last one was used as a test set. This procedure was repeated so that every subset was selected as test data once. The cross-validated Root-Mean-Square Error (RMSECV) was then computed.

External validation was performed by splitting the initial dataset into training and test data. After classifying polyphenols by ascending BLD, approximately 3 out of 4 molecules were kept in the training set. This allowed to build a reliable test set of 8 molecules made of ~23% of the initial dataset (Levet et al., 2013). Several external validation criteria may be used to assess QSAR predictivity and robustness (Chirico and Gramatica, 2011, 2012). Here, the classical squared correlation coefficient *Q*^2^_F1_ (Schüürmann et al., 2008) which is advocated in the OECD guidelines (OECD, 2007) was used and is expressed as:

(2) $$Q_{F1}^{2}=1-\frac{{\sum_{i=1}^{n_{test}}\left(y_{i}-{\hat{y}}_{i}\right)}^{2}}{{\sum_{i=1}^{n_{test}}\left(y_{i}-{\bar{y}}_{train}\right)}^{2}}$$

where *y*_*i*_ and $\hat{y_{i}}$ are the observed and the predicted activity, respectively of the test set. ${\bar{y}}_{train}$ is the mean observed BLD of the training set. The used acceptance values of *Q*^2^_F1_ are 0.70.

Finally, the Y-Randomization method was used to exclude the possibility that the model performance is due to chance correlation (Roy et al., 2009). BLD values were randomly permuted and new QSAR was developed using the same descriptors as included in the unrandomized model. The $R_{p}^{2}$ parameter allows comparing the performance of the randomized and unrandomized models, through their determination coefficients $R_{T}^{2}$ and *R*^2^, respectively. $R_{p}^{2}$ parameter is defined as:

(3) $$\begin{array}{r} R_{p}^{2}=R^{2}\times \sqrt{R^{2}-R_{T}^{2}} \end{array}$$

$R_{p}^{2}$ > 0.5 ensure that models are not obtained by chance.

Developed QSAR models also require the definition of the corresponding applicability domain (AD) for estimating the reliability in the prediction of a new molecule (OECD, 2004). Predicted activity for only those compounds that fall into this domain may be considered reliable. In this work, the euclidean distance between the centroid and each polyphenol of the training set was computed for each descriptor involved in the QSAR models. The AD was defined as the limit distance values including 95% of the polyphenols.

#### Determination of Bacterial Cells Surface Properties

The hydrophobicity and the electron-donor/electron-acceptor character, i.e., Lewis acid/base of cell surface of the 6 bacterial strains was evaluated by Microbial Adhesion To Solvents (MATS) according to the method proposed by Bellon-Fontaine et al. (1996). This partitioning is based on the comparison on one hand between microbial cell affinity to chloroform (a polar monoacidic electron accepting solvent) and hexadecane (an apolar solvent) and on the other hand between ethyl acetate (a strong electron donor solvent) and hexane (another apolar solvent). The polar solvent can be an electron acceptor or an electron donor, but both solvents must have similar van der Waals surface tension components.

Bacterial cells were harvested by centrifugation at 7,000 × g for 10 min at 4°C, washed twice in physiological water (150 mmol.L^−1^ NaCl) and resuspended to OD_400nm_ = 0.8, 0.4 mL of the solvent under investigation was added to 2.4 mL of cell suspension. The two phase system was mixed with a vortex for 2 min and allowed to separate for 15 min to ensure complete separation of the two phases before sampling. One milliliter was carefully removed from the aqueous phase and the OD_400nm_ was measured. The percentage of microbial adhesion to solvent was calculated as follows: (1-OD/OD_0_) × 100 where OD_0_ and OD are the optical density measured at 400 nm of the bacterial suspension before and after mixing, respectively. This determination of MATS was repeated 3 times with each solvent for each bacterial strain.

## Results

### Effect of Polyphenols on Bacterial Growth

**Figure 1:**
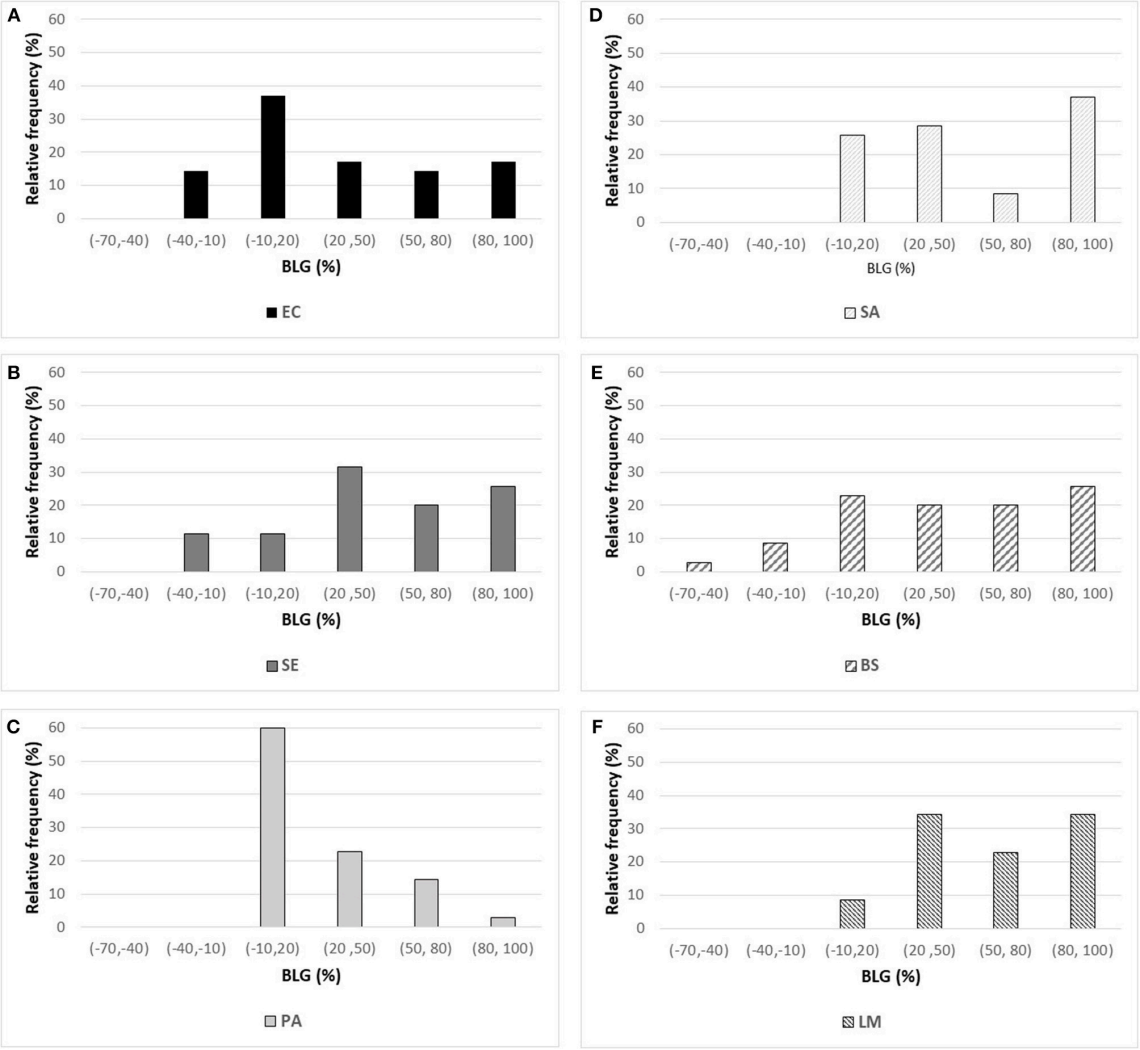
Histogram of BLD experimental values measured for the 35 polyphenols against three Gram-negative bacteria [on the left: **(A)** *E. coli* ATCC25922 (EC), **(B)** *S*. Enteritidis E0220 (SE), and **(C)** *P. aeruginosa* ATCC27853 (PA)] and three Gram-positive bacteria [on the right: **(D)** *S. aureus* CNRZ3 (SA), **(E)** *B. subtilis*ATCC6633 (BS), and **(F)** *L. monocytogenes* ATCC19115 (LM)].

The effects of the 35 polyphenols at a 1 g L^−1^ concentration on the growth of the 6 microbial strains tested (Table 1) are presented in Figure 1. BLD experimental values ranged from −67.8% up to 100% indicating the existence of completely antagonistic effects: molecules annihilated the bacterial growth (BLD about 100%) while others favored it (negative BLD).

The standard deviation (SD) has been calculated from the triplicates performed for each experiment (Table 1). From these results, we determined a global estimation of SD and the corresponding 95% confidence interval of BLD obtained for each bacterial strain (Table 1). These values indicated that BLD results were given at approximately ±20% (95% confidence interval).

As stated in Table 1, the molecular weights of the 35 polyphenols tested at a 1 g L^−1^ concentration ranged from 168 to 611 g.mol^−1^ (mean ± SD (standard deviation) (*n* = 35) = 303 ± 125 g.mol^−1^). Examination of the distribution of molecular weights allowed to divide polyphenols into 3 groups: 8 polyphenols had a <200 g.mol^−1^ molecular weight (i.e., slightly lower), 20 polyphenols had a molecular weight between 200 and 400 g.mol^−1^, and finally 7 polyphenols had a molecular weight exceeding 400 g.mol^−1^ (up to 611 g.mol^−1^). As a consequence, the molar concentrations of the 35 polyphenols corresponding to a 1 g L^−1^ concentration ranged thus from 1.64 to 5.95 mmol.L^−1^. Since most of QSAR studies are performed at a fixed molar concentration, in order to have a fixed number of molecules to bacterial cells ratio, it was first checked whether the intensity of BLD was influenced by these differences in molar concentrations. Therefore, the mean BLD against *E. coli* was also determined at 4 mmol.L^−1^ for seven polyphenols chosen so as to well-represent the whole dataset in terms of molecular weight (Table 2). A Student's *t*-test was used to compare the mean BLD values at 1 g L^−1^ and 4 mmol.L^−1^. No statistically significant difference was observed between the two means (*p* < 0.05). The biggest difference, although not statistically significant, was obtained for ethyl-3,4-dihydroxy-cinnamate (WDK) from which molecular weight (208 g.mol^−1^) belongs to the most represented group in the dataset. Moreover, no statistically significant correlation between mean BLD values (at 1 g L^−1^ and 4 mmol.L^−1^) and the corresponding molecular weight of the 7 polyphenols was found since the correlation coefficients *R* were −0.26 and −0.49, respectively.

**Table 2:** Comparison of the antibacterial properties of 7 polyphenols determined at 1 g L^−1^ and 4 mmol.L^−1^ against *E. coli* ATCC25922.

**Shortened InChikey ID**	**Polyphenol**	**Molecular weight (g.mol^**−1**^)**	**Concentration equivalent to 1 g L^**−1**^ (mmol.L^**−1**^)**	**BLD% (average) at 1 g L^**−1**^**	SD^*^	**BLD% (average) at 4 mmol.L^**−1**^**	SD^*^
HGE	2,4-dihydroxy-cinnamic acid	180	5.55	−7.8	4.0	−11.0	4.7
WDK	Ethyl-3,4-dihydroxy-cinnamate	208	4.80	34.8	3.1	15.8	18.4
HDP	5,7-dihydroxy-4-propylcoumarin	220	4.54	−1.8	9.8	0.1	8.7
TTY	Caffeic acid 1,1-dimethylallyl ester	248	4.02	17.5	2.5	24.2	3.8
CXQ	Taxifolin	304	3.29	18.1	3.1	11.4	5.3
CWV	Chlorogenic acid	354	2.82	−4.2	1.1	−15.3	5.9
YDD	(-)-Chicoric acid	474	2.11	−17.1	6.1	−3.0	3.8

* *SD is the standard deviation determined from triplicates*.

Examination of the different histograms of Figure 1 allows to compare the effects of the 35 polyphenols tested on each of the 6 bacterial strains. Overall, *P. aeruginosa* was poorly sensitive to polyphenols with 60% of the molecules leading to BLD values comprised between −10 and 20% (Figure 1C). For this bacterial strain, the best antimicrobial effect was obtained with epigallocatechin gallate (WMB, 74.7% BLD) and caffeic acid (QAI, 84% BLD). To a lower extent, the same behavior was observed for *E. coli* (Figure 1A) with 51.4% polyphenols having BLD lower than 20%. Conversely, *L. monocytogenes* (Figure 1F) was greatly affected by the tested phenolic compounds with 54.3% of polyphenols characterized by BLD above 50%. Satisfying antimicrobial effects were also observed against *S. aureus* (Figure 1D) and *B. subtilis* (Figure 1E) with 45.7% of polyphenols exhibiting BLD above 50%. Among the Gram-negative bacteria, *S*. Enteritidis (Figure 1B) was the bacterial strain the most affected by the phenolic compounds with also 45.7% of BLD values above 50% (against 31.4% for *E. coli* and 17.1% for *P. aeruginosa*).

By considering the negative BLD values (BLD<–10%), 10–15% of polyphenols favored the growth of *E. coli, S*. Enteritidis, and *B. subtilis*. Moreover, one polyphenol showed a very negative BLD value about −70% indicating a very important growth-promoting effect: myricitrin dihydrate (DCY) with respect to *B. subtilis* (Figure 1E). The presence of amine or glycosyl conjugated groups to polyphenols can promote the growth of the cultured bacteria by supplying nutrients, respectively nitrogen or fermentable sugars. This could explain the *B. subtilis* growth-promoting effect of myricitrin which is glycosylated. However, this stimulatory effect of growth by certain phenolic compounds was not observed for all bacterial strains studied.

Among the three stilbenes studied, two molecules exhibited high antibacterial effect against five of the six bacterial strains tested: resveratrol (LUK) and pinosylvin (YCV). The latter was very active against Gram-positive bacteria but ineffective against *P. aeruginosa*. Resveratrol appeared the most interesting polyphenol tested with relatively high BLD values: 60.2% BLD against *P. aeruginosa*, 75.2% against *B. subtilis*, and 100% for the other 4 bacterial strains. The antibacterial activity of pinosylvin against *S. aureus* was already reported by Plumed-Ferrer et al. (2013). However, the last stilbene, rhapontin (GKA), did not have any significant antimicrobial effect with BLD values between −15.0 and 16.7% depending on the bacterial strain considered. Unlike, pinosylvin and resveratrol, rhapontin is a monoglycosylated stilbene. Interestingly, Kim et al. (2010) reported a 4–16 times higher antibacterial activity of rhapontigenin, the aglycone part of rhapontin, than rhapontin against *E. coli, P. aeruginosa*, and *S. aureus*. These authors suggested that glycosylation may have reduced antibacterial activity of rhapontin namely by reducing its number of free hydroxyl groups and lipophilicity or by increasing steric hindrance, all factors involved in the antibacterial activity of plant phenolics.

Among the cinnamic acids tested, only cinnamyl-3,4-dihydroxy-α-cyanocinnamate (XGH) had an interesting antimicrobial profile with 100% BLD in the case of Gram-positive bacteria and also good performance against Gram-negative ones with BLD values between 59.3 and 82.1%. Interestingly, caffeic acid 1,1-dimethylallyl ester (TTY) antibacterial activity was always significantly higher against the 3 Gram-positive bacterial strains tested than that of caffeic acid (QAI). Conversely, the antibacterial activity of this ester of caffeic acid was unchanged or decreased for the 3 Gram-negative bacterial strains tested. Esterification of caffeic acid by 1,1 dimethyl allyl group resulted in an increase of its calculated log D pH 5.5 value from 0.04 to 2.79. These observations are consistent with those reported by Andrade et al. (2015) who finely tuned the hydrophobicity of caffeic acid by preparing esters of caffeic acid with varying alkyl ester side chain length. They systematically studied the antibacterial action of caffeic acid and these caffeic acid alkyl esters against a *S. aureus* strain and an *E. coli* strain and came to the conclusion that longer alkyl side chains were more effective against the Gram-positive bacterium, while medium length alkyl side chain caffeic acid esters were more effective against the Gram-negative bacterium which was also far less susceptible to caffeic acid and its esters.

In general, the bacterial strains the most affected by benzoic acids were *B. subtilis* and *S*. Enteritidis. Butyl gallate (XOP) was the most active benzoic acid, inhibiting all the bacterial strains tested except *P. aeruginosa*. The *in vitro* antibacterial activity against pathogenic bacteria of phenolic (cinnamic or benzoic) acids was already reported in the literature, especially for sinapinic acid (Engels et al., 2012) and for gallic and ferulic acids (Borges et al., 2013). Butyl p-hydroxybenzoate (or butylparaben) used as a preservative in foods and cosmetics also exhibits bactericidal effect against *S. aureus* (Quévrain et al., 2009).

Among the eleven flavonoids of the dataset, only epigallocatechin gallate (WMB) was active against the bacterial strains tested, except *E. coli*. This observation is in accordance with the results of Yoda et al. (2004) who showed an inhibition of the growth of various strains of *Staphylococcus* by epigallocatechin gallate with minimum inhibitory concentrations (MIC) ranging from 0.05 to 0.1 g L^−1^. The presence of gallic or galloyl moieties should promote the antibacterial activity of epigallocatechin gallate by inducing damages to bacterial membrane (Ikigai et al., 1993).

Gram-positive bacteria, especially *L. monocytogenes* and *S. aureus*, were more sensitive than Gram-negative ones to coumarins. 3',5'-dihydroxyflavone (MCC) was the most active coumarin reaching 39.6% BLD against *B. subtilis*, 49% BLD against *E. coli* and BLD values above 70% for the four other strains. For *S. aureus* and *B. subtilis*, a decreasing antimicrobial activity was observed: 5,7-dihydroxy-4-phenylcoumarin (HUQ) >5,7-dihydroxy-4-propylcoumarin (HDP) >5,7-dihydroxy-4-methylcoumarin (QNV). This evolution suggested that the substitution of the phenyl moiety by a propyl or a methyl one was deleterious for the antibacterial effect. This negative effect was also observed against the last Gram-positive bacteria *L. monocytogenes* when substituting the phenyl by the propyl moiety with a decrease of BLD from 93.5 down to 46.9%. However, antibacterial activity still remained high in the case of 5,7-dihydroxy-4-methylcoumarin (QNV).

Finally, the two naphtoquinones studied had very interesting antibacterial profile. 5,8-dihydroxy-1,4-naphthoquinone (RQN) was very active (BLD>89.6%) against the bacterial strains tested but in a lower extent against *P. aeruginosa* (BLD 35%). 2,3-dichloro-5,8-dihydroxy-1,4-naphthoquinone (UVE) had similar antimicrobial properties but was significantly less active against *E. coli* (BLD 34%) and *P. aeruginosa* (BLD −5.3%). Medina et al. (2006) also reported bactericidal effect against *S. aureus* of naphtoquinones which would act as oxidative agents. Naphtoquinones were found to inhibit potential efflux pumps which, in the case of Gram-negative bacteria, are particularly involved in their resistance to most of natural antimicrobial products (Kuete et al., 2011).

### QSAR Modeling

The wide range of the experimental values measured for the antibacterial activity of the 35 polyphenols depended on the bacterial strain tested and suggested the involvement of different types of mechanism of action against bacteria, in accordance with the literature. Nevertheless, and despite also the variability observed in the BLD measurements, the whole polyphenol dataset was exploited so as to develop QSAR models. The objectives were to predict BLD property regardless of the type of polyphenols as well as the mechanisms of toxic action involved and thus to highlight the polyphenol structural characteristics that should explain their antibacterial activity.

QSAR models were developed for each tested bacterial strain according to the methodology described in part 2.3. The predictive power and robustness of the models developed were assessed by internal and external validation techniques. Note that the molecules of the test set depended on the bacterial strain considered and were chosen so as to be representative of the whole BLD range. From the experimental variability characterizing BLD measurements, Root-Mean-Square Errors (RMSE) <25% were considered as acceptable values to assess model quality.

No reliable QSARs were obtained for *P. aeruginosa* and *L. monocytogenes* strains (results not shown). This result was not surprising considering the BLD values characterizing the activity of the 35 polyphenols against these two bacterial strains. In the case of *P. aeruginosa*, most of polyphenols had no significant effect on its bacterial growth (i.e., 60% of polyphenols modified *P. aeruginosa* growth by <20%) while they were very efficient against *L. monocytogenes* (i.e., 54.3% of polyphenols inhibited its growth by more than 50%). This poor statistical distribution of BLD values (Figures 1C,F, respectively) did not allow obtaining satisfactory models.

For the other four strains, the best predictive QSAR models including five to six descriptors and their corresponding applicability domain are presented in Table 3. The values of the explicative variables are provided in Table 4 for the 35 polyphenols. The indicators that reflected both the quality and predictive performance of each model are reported in Table 5. The determination coefficients *R*^2^ were comprised between 0.783 for *B. subtilis* model and 0.867 for *S. aureus*, which indicated satisfying model fitting quality. Moreover, as expected from robust models, *Q*^2^_F1_ were above 0.7 and RMSE values from training (RMSE), cross-validation (RMSECV) and test (RMSEP) sets were similar for each model. RMSE values ranged from 14.8 to 21.9% for *E. coli, S*. Enteritidis and *S. aureus*. They reached slightly higher values in the case of *B. subtilis* (20.8–26.7%) but these remained completely in accordance with the experimental variability observed in BLD measurements. Moreover, the model randomization method led to *R*^2^p > 0.6 also proving the robustness of the developed QSAR models.

**Table 3:** QSAR models and their applicability domain for the prediction of BLD depending on the bacterial strain considered: *E. coli* ATCC25922, *S*. Enteritidis E0220, *B. subtilis* ATCC6633, and *S. aureus* CNRZ3.

**QSAR**	**Applicability domain**
BLD *E. coli* = −26.56 −1,360*v_μ = –(I+A)/2 +80.23*HOMO-1(eV) −8.66* Dipole moment −203.91*Minimum atomic charge (Mulliken) +0.75*-0.05 < S < 0 +9.54*Rotatable bonds	−0.3584 < v_μ <–0.2446 −6.643 < HOMO-1 (eV) <–5.360 Dipole moment <9.739 −0.642 < Minimum atomic charge (Mulliken) <–0.469 “−0.05 <S < 0” <84.2% 0.508 < Rotatable bonds <1.027
BLD *S*. Enteritidis = −4.066 −768.55*v_μ = –(I+A)/2 +0.37*Polarisability −108.62*Maximum atomic charge(Mulliken) +0.35*-0.05 < S < 0 −224.88*Molecular complexity	−0.3584 < v_μ <–0.2446 Polarisability <296.92 0.392 <Maximum atomic charge (Mulliken) <0.901 “−0.05<S 0” <84.2% 0.508 < Molecular complexity <1.027
BLD *B. subtilis* = 271.27 +12.37*v_N- −1,122*w_λ_E +20.33*Log D pH (5.5) −2.30*S >0.05 +16.24*H-Donors −287.38*Molecular complexity	5.798 < v_N- <14.119 0.113 < w_λ_E <0.271 −1.49 < Log D pH(5.5) <4.58 “S > 0.05” <20.8% H-Donors <9 0.508 < Molecular complexity <1.027
BLD *S. aureus* = −252.88 + 1665*X5A +18.05*RDCHI +22.93*Log D pH (5.5) −25.55*LUMO (eV) +3.69*Dipole moment −1.23*S < −0.1	0.059 < X5A < 0.091 1.409 < RDCHI <5.123 −1.49 < Log D pH(5.5) <4.58 −4.070 < LUMO (eV) <–1.749 Dipole moment <9.739 “S <–0.1” <44.3%

**Table 4:** Descriptors involved in the developed QSAR models.

**Shortened InChIKey**	**v_μ = –(I+A)/2**	**HOMO-1 (eV)**	**Dipole moment**	**Minimal atomic charge (Mulliken)**	**0.05 < S < 0**	**Rot. bonds**	**Polarisab**.	**Maximal atomic charge (Mulliken)**	**Molecular complexity**	**v_N-**	**w_λ_E**	**Log D pH (5.5)**	**S > 0.05**	**H-Donors**	**X5A**	**RDCHI**	**LUMO (eV)**	**S < −0.1**
APH	−0.3300	−6.166	2.258	−0.546	49.8	3	74.61	0.727	0.615	9.901	0.190	1.90	2.5	2	0.086	2.414	−2.434	0.0
CUF	−0.3294	−6.417	3.683	−0.545	77.8	2	61.12	0.746	0.634	9.960	0.193	1.54	0.0	2	0.086	2.282	−2.225	0.0
CWV	−0.2938	−6.268	6.040	−0.589	4.3	5	158.11	0.714	0.809	9.916	0.176	−2.48	8.4	6	0.094	2.642	−3.349	0.0
CXQ	−0.2838	−5.871	2.530	−0.592	0.0	1	138.80	0.494	0.852	12.113	0.226	1.64	12.7	5	0.091	3.057	−2.923	0.0
DCY	−0.2792	−5.853	1.461	−0.607	0.0	3	183.81	0.738	0.935	9.867	0.201	0.68	27.1	8	0.072	2.243	−3.062	0.0
FXN	−0.3033	−5.936	3.367	−0.576	42.4	1	94.38	0.448	0.834	9.916	0.194	2.94	0.0	3	0.082	2.732	−3.090	0.0
GKA	−0.2642	−5.686	2.983	−0.568	0.0	6	182.48	0.769	0.824	10.956	0.227	0.37	23.8	6	0.074	3.102	−2.449	0.0
GZS	−0.2638	−5.953	7.455	−0.583	0.0	7	400.85	0.753	0.951	11.783	0.205	0.00	17.3	8	0.074	3.221	−3.057	0.0
HDP	−0.3235	−6.076	7.900	−0.501	20.0	2	92.69	0.584	0.798	9.658	0.187	2.15	0.0	2	0.072	2.457	−2.635	0.0
HGE	−0.3231	−6.204	6.870	−0.511	0.0	2	63.15	0.650	0.627	8.133	0.177	−0.26	2.6	3	0.071	3.216	−2.875	0.0
HUQ	−0.3107	−6.104	7.794	−0.497	60.4	1	119.43	0.587	0.826	9.620	0.183	3.06	0.0	2	0.069	2.677	−2.832	0.0
IKG	−0.2651	−5.786	5.516	−0.645	45.5	6	257.22	0.763	0.973	10.451	0.201	−0.30	0.0	10	0.077	3.513	−3.012	0.0
IKM	−0.2862	−5.821	1.953	−0.631	0.0	1	97.92	0.428	0.856	9.378	0.200	1.58	6.8	6	0.086	3.732	−2.964	0.0
KJX	−0.2830	−5.758	3.668	−0.591	50.6	1	139.59	0.493	0.856	12.164	0.229	1.29	9.4	6	0.073	3.014	−2.967	0.0
LUK	−0.2847	−5.498	3.180	−0.466	100.0	2	88.31	0.389	0.607	10.169	0.223	2.84	0.0	3	0.088	3.096	−2.463	0.0
MCC	−0.3084	−5.941	3.443	−0.530	46.9	1	94.84	0.449	0.775	9.632	0.186	2.73	0.0	2	0.073	3.091	−3.034	0.0
NYS	−0.2988	−5.797	4.173	−0.562	31.4	4	105.29	0.773	0.699	9.490	0.177	3.79	0.0	2	0.070	3.189	−3.230	0.0
OVS	−0.2803	−6.13	6.560	−0.628	56.6	4	194.68	0.737	0.938	9.717	0.192	0.33	0.9	8	0.077	3.981	−3.334	0.0
QAI	−0.3269	−6.359	6.312	−0.510	0.0	2	62.92	0.651	0.611	8.100	0.176	0.04	21.9	3	0.073	2.620	−2.999	0.0
QNV	−0.3273	−6.079	7.875	−0.501	40.8	0	67.95	0.588	0.765	9.535	0.189	1.65	0.0	2	0.073	2.445	−2.603	0.0
RNV	−0.3315	−6.193	2.068	−0.541	59.8	2	61.51	0.745	0.602	9.622	0.190	1.58	0.0	2	0.069	2.584	−2.472	0.0
RQN	−0.3719	−6.68	0.155	−0.542	43.9	0	60.87	0.477	0.795	4.585	0.067	1.95	10.0	2	0.075	3.333	−4.564	0.0
SEB	−0.2714	−5.758	5.268	−0.580	12.2	4	286.49	0.772	0.940	12.456	0.237	2.26	0.0	5	0.073	2.571	−2.983	43.4
TTY	−0.2919	−5.957	2.834	−0.559	20.9	5	126.76	0.706	0.580	10.610	0.196	2.79	0.0	2	0.080	2.406	−2.728	0.0
TXG	−0.3173	−5.787	4.546	−0.548	88.3	3	88.65	0.727	0.615	10.778	0.204	1.99	0.0	3	0.063	3.466	−2.136	0.0
UBQ	−0.3065	−6.149	5.475	−0.610	23.7	8	181.12	0.754	0.889	7.527	0.159	3.14	0.0	3	0.076	2.449	−3.309	0.0
UVE	−0.3662	−7.036	1.818	−0.531	46.2	0	72.16	0.509	0.843	4.090	0.061	2.76	11.8	2	0.067	2.538	−4.760	0.0
VHN	−0.3273	−6.044	6.480	−0.524	21.7	1	69.30	0.678	0.785	10.588	0.200	−0.59	0.0	3	0.084	2.344	−2.074	0.0
WDK	−0.3128	−6.176	3.503	−0.554	21.2	4	86.68	0.698	0.559	9.116	0.183	2.18	0.0	2	0.075	4.037	−2.714	43.0
WMB	−0.2433	−5.334	3.758	−0.554	11.5	4	298.52	0.735	0.864	16.641	0.286	1.83	13.2	8	0.068	4.933	−2.319	0.0
XGH	−0.2869	−5.922	1.570	−0.549	27.1	6	211.21	0.751	0.659	9.776	0.173	2.89	0.0	2	0.065	4.762	−3.408	8.7
XOP	−0.3140	−5.806	4.427	−0.546	40.2	5	102.37	0.727	0.619	10.855	0.203	2.48	11.7	3	0.067	5.392	−2.163	0.0
XQD	−0.2801	−5.969	2.505	−0.563	18.5	1	101.84	0.751	0.947	10.780	0.229	2.54	0.0	3	0.066	4.319	−2.608	44.3
YCV	−0.2940	−5.536	1.180	−0.467	19.0	2	85.96	0.390	0.592	9.664	0.205	3.13	0.0	2	0.071	5.474	−2.575	0.0
YDD	−0.2722	−6.004	13.707	−0.591	10.2	11	224.45	0.722	0.789	10.995	0.201	−2.54	0.0	6	0.064	4.972	−3.486	0.0

**Table 5:** Indicators of the fitting quality (*R*^2^, RMSE) and the robustness (RMSECV, RMSEP, *Q*^2^_F1_, and *R*^2^p) of the QSARs developed for BLD prediction of *E. coli, S*. Enteritidis, *B. subtilis*, and *S. aureus*.

**Bacterial strain**	***E. coli*** ATCC25922	***S*. Enteritidis** E0220	***B. subtilis*** ATCC6633	***S. aureus*** CNRZ3
Number of variables in the QSAR	6	5	6	6
*R*^2^	0.825	0.786	0.783	0.867
RMSE	19.1	18.3	22.5	14.8
RMSECV	21.8	21.9	26.7	16.7
RMSEP	17.8	15.8	20.8	15.7
*R*^2^p	0.665	0.623	0.609	0.730
*Q*^2^_F1_	0.817	0.741	0.712	0.814

By considering polyphenols exhibiting high and low antimicrobial activity against the four bacterial strains, a comparative model-by-model study of the influence of the explicative descriptors has been made in order to identify structure-activity links. Chlorogenic acid (CWV), myricitrin dihydrate (DCY), and rhapontin (GKA) had no significant antibacterial effect with respect to the four bacterial strains considered while resveratrol (LUK), 5,8-dihydroxy-1,4-naphthoquinone (RQN) and butyl gallate (XOP) exhibited high antibacterial activity.

In the case of *E. coli*, the most discriminating parameter between the two types of antibacterial behavior was the electrostatic potential-based parameter “−0.05 <S <0” (Table 3). The largest surface percentages in the zone “−0.05 <S <0” corresponded to hydrophobic molecules (LUK, RQN, and XOP). The positive coefficient of this parameter in the BLD model with *E. coli* thus indicated that the antibacterial activity against *E. coli* increased with polyphenol lipophilicity, in accordance with the literature (Sikkema et al., 1995). Both electronic (HOMO(-1), v_μ) and electric charge [S, dipole moment, minimum atomic charge (Mulliken)] properties of polyphenols were important for the explanation of BLD with *E. coli*. The positive coefficient of the “rotatable bonds” descriptor showed that rigid molecules should be less effective.

The model developed for the second Gram-negative bacteria, *S*. Enteritidis, included very similar explicative variables indicating that the mechanisms of toxic action against *S*. Enteritidis were very similar to the ones against *E. coli*. The same observations can be made about the lipophilicity role (“−0.05 <S <0”) and the importance of electric charge and electronegativity in the antibacterial activity. The molecular complexity (CWV, DCY, and GKA) appeared to disadvantage the antibacterial effect (Table 3). This might be due to steric hindrance that may limit interactions between the polyphenol and the bacteria cell wall. However, esterification of quinic acid by caffeic acid in the case of chlorogenic acid (CWV) or glycosylation in the cases of myricitrin and rhapontin may also have resulted in a lower lipophilicity of these molecules compared to similar polyphenols and thereby in a decrease of their antibacterial activity.

For *B. subtilis*, the less active molecules are generally hydrophilic molecules (low Log D pH (5.5) and high S > 0.05 values). The most active polyphenols (LUK, RQN, and XOP) were less complex with two or three H-donors sites, less hydrophilic with Log D values comprised between 2 and 3 that may favor the passage of these molecules through the bacteria membrane. Once again lipophilicity, molecular complexity, and electronic properties of polyphenols (v_N- and w_λ_E) appeared important to describe BLD of *B. subtilis*.

The same structure of QSAR was obtained for *S. aureus* since including Log D pH (5.5), S < −0.1, LUMO and dipole moment as explicative variables. The negative coefficient of LUMO energy suggests that highly electrophilic polyphenols had high antibacterial activity. The topological and connectivity indices (RDCHI and X5A) also involved in the model were difficult to interpret in the absence of knowledge about polyphenol receptors on bacteria surface.

**Figure 2:**
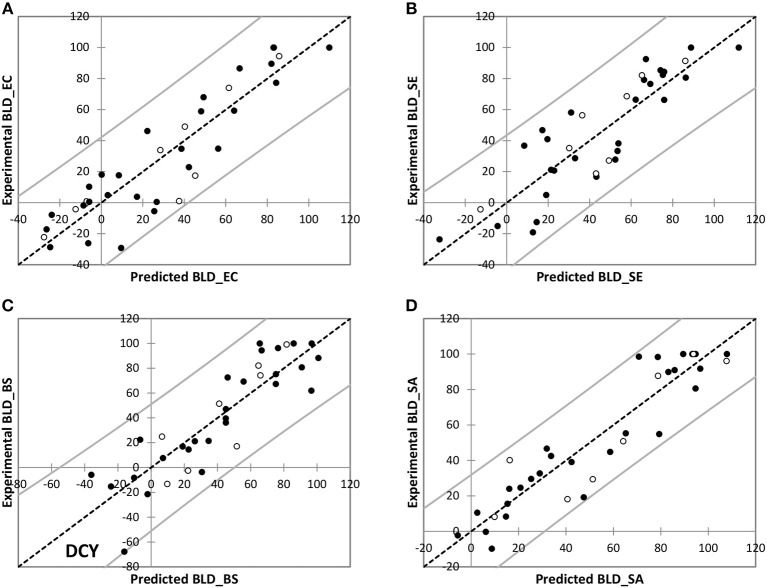
Experimental *vs*. predicted BLD values for training (•) and test (◦) polyphenols for *E. coli* ATCC25922 **(A)**, *S*. Enteritidis E0220 **(B)**, *B. subtilis* ATCC6633 **(C)**, and *S. aureus* CNRZ3 **(D)**.

Modeling results are presented in Figure 2. Predicted and experimental values of BLD were considered similar with respect to the experimental variability. This was observed for both training and test sets whatever the bacterial strain considered. In the case of *B. subtilis*, an outlier has been highlighted that may be explained by its specific and single high bacterial growth-stimulating effect: myricitrin dihydrate (DCY) exhibited BLD about −67.8% but the predicted value was −16.1%. This prediction was not satisfactory since the corresponding residual about −51.6% was too large; however, the bacterial growth-stimulating behavior was well-predicted by the model. In spite of such important residual, statistical indicators remained quite satisfying for the QSAR fitting quality (Table 5).

### Bacterial Cells Surface Properties as Estimated by Microbial Adhesion to Solvents

The most commonly reported mechanism of action of phenolics against bacteria is based on their accumulation at the surface of bacteria (Negi, 2012). This accumulation depends on interactions between phenolics and the cell wall of bacteria. Therefore, the surface properties of the 6 strains of bacterial cells were investigated by determining the microbial adhesion to water-solvent interfaces of these cells (Table 6). Comparison of the adhesion to water-hexadecane or water-hexane and to water-chloroform or water-ethyl acetate of the cells of these 6 bacterial strains revealed that *S. aureus* CNRZ3 and *L. monocytogenes* ATCC19115 cells surface strongly differed from the surfaces of the 4 other cell types. The surface of the cells of both bacterial strains had the highest affinity for chloroform (85 ± 4 and 86 ± 2%, respectively), an acidic solvent with electron-acceptor properties: it can thus be proposed that it results from a basic or electron-donating property of these bacterial cells. This observation is consistent with conclusions of a MATS study of 22 *L. monocytogenes* isolates (Lee et al., 2017): they also reported the electron-donating properties of the surface of theses bacterial cells. Microbial adhesion to the n-alkanes investigated in this study of bacteria of both strains were far lower: 47.3 ± 3.7 and 63.8 ± 1.6% for hexane, 33.4 ± 0.4, and 36.3 ± 8.6% for hexadecane, respectively. Surface of *S. aureus* CNRZ3 and *L. monocytogenes* ATCC19115 cells can thus be considered hydrophilic. However, the affinity for hexadecane of the 4 other cell types was always <3.7 and 0.6%, respectively. This suggests that cells surfaces of both strains contains highly hydrophilic but also hydrophobic zones, which would explain their capacity to adhere both to hydrophilic and hydrophobic surfaces. Their surface can thus be considered as amphiphilic. However, the affinity of *S. aureus* CNRZ3 cells to ethyl acetate-water interfaces (90.0 ± 0.3%) was far higher than that of *L. monocytogenes* ATCC19115 cells (19.4 ± 5.1%). This indicates electron-accepting properties of *S. aureus* CNRZ3 cells surface unlike *L. monocytogenes* ATCC19115 cells surface since ethyl acetate is an electron donor solvent.

**Table 6:** Adhesion of bacterial cells to solvent-water interfaces (*n* = 3).

**Solvent**	**Percentage (%) adhesion of bacterial cells**					
	***E. coli*** **ATCC25922**	***S*. Enteritidis** **E0220**	***P. aeruginosa*** **ATCC27853**	***B. subtilis*** **ATCC6633**	***S. aureus*** **CNRZ3**	***L. monocytogenes*** **ATCC19115**
Chloroform	58 ± 3^a^	67 ± 5^b^	23 ± 1^c^	27 ± 1^d^	85 ± 4^e^	86 ± 2^e^
Hexadecane	2.3 ± 0.1^a^	0.3 ± 0.5^b^	3.7 ± 1.5^a^	0.0 ± 2.4^b^	33.4 ± 0.4^c^	36.3 ± 8.6^c^
Hexane	0.0 ± 0.5^a^	0.0 ± 1.1^a^	0.0 ± 0.4^a^	0.6 ± 0.9^a^	47.3 ± 3.7^b^	63.8 ± 1.6^b^
Ethyl acetate	26.5 ± 1.1^a^	21.0 ± 1.2^b^	20.0 ± 0.8^b^	13.9 ± 4.7^c^	90.0 ± 0.3^d^	19.4 ± 5.1^b^, ^c^

a−e *Means within rows with different superscript letters are significantly different (P < 0.05)*.

The other Gram-positive bacterial strain considered, *B. subtilis* ATCC6633, had no affinity for hexane and hexadecane and a far lower affinity for chloroform (27 ± 1%) as well as a low affinity for ethyl acetate (13.9 ± 4.7%). It can thus be concluded that its surface properties were the most hydrophilic among the 3 Gram-positive bacterial strains.

The adhesion to solvent properties of *P. aeruginosa* ATCC27853 cells surface were quite similar to those of *B. subtilis* ATCC6633 cells surface. Its surface can thus also be considered as highly hydrophilic. While the surface of the 3 Gram-negative bacterial cells all had a low hydrophobicity based on their absence or low affinity for hexane-water or hexadecane-water interfaces (i.e., always <3.7%), *E. coli* ATCC25922 and *S*. Enteritidis E0220, had a similar affinity for chloroform (about 60%): their affinity for chloroform was thus significantly higher than that of *P. aeruginosa* ATCC27853 (23 ± 1%). This higher affinity of *E. coli* ATCC25922 and *S*. Enteritidis E0220 for chloroform suggests a Lewis-base character and electron-donating properties of their surface.

It can thus be concluded from these MATS studies that *E. coli* ATCC25922 and *S*. Enteritidis E0220 surfaces present an intermediate polarity between the amphiphilic surface of the 2 Gram-positive *S. aureus* CNRZ3 and *L. monocytogenes* ATCC19115 bacterial cells and the polar surface of *P. aeruginosa* ATCC27853 and *B. subtilis* ATCC6633 cells.

## Discussion

### Effect of Polyphenols on Bacterial Growth

In many studies, the antibacterial activity of molecules is evaluated by measuring their minimal inhibitory concentrations (MIC): the minimal concentration which prevents bacterial growth. A good example is the study of Taguri et al. (2006) who determined the MICs of 22 polyphenols against 26 species of bacteria. The MICs of bacterial growth inhibitory polyphenols varied between 0.067 and 3.200 g L^−1^. In the present study, the growth of 6 bacterial strains was monitored over 24 h in the presence of 1 g L^−1^ of each of the 35 tested polyphenols. As expected, after 24 h incubation, a significant decrease (BLD > 20%) of bacterial growth in the presence of 1 g L^−1^ of polyphenols was observed for 69.5% of assays. This is consistent with the mean MIC value against 26 species of bacteria ranging between 492±347 and 2,782±602 mg L^−1^ (i.e., close to 1 g L^−1^) reported for 22 polyphenols by Taguri et al. (2006). However, this also corresponded to a non-significant decrease of bacterial growth for 30.5% of observations and even to a significant bacterial growth-promoting effect (BLD > −10%) for 8.6% of observations. Indeed, one must keep in mind that when a medium like MHB is supplemented with 1 g L^−1^ of polyphenol, it cannot be excluded that the added polyphenol might promote bacterial growth although, by definition, this kind of observation is not reported in studies based on MIC determination. This bacterial growth-promoting effect could result from the action of bacterial enzymes on polyphenols: interestingly, about one half of growth-promoting effects were observed with O-glycosylated polyphenols (diosmin, rhapontin) and with chlorogenic acid which is an ester of quinic acid with caffeic acid: the growth-promoting effect of these polyphenols in MHB might result from the release of glucose or quinic acid by enzymatic hydrolysis. However, some other non-esterified or glycosylated polyphenols (e.g., silibinin, wedelolactone or chicoric acid) also promoted bacterial growth: in such cases, a cleavage of C-C bonds by bacterial enzymes cannot be excluded. Such a cleavage of flavonoids by gut microbiota microorganisms has been suggested by several authors and recently reviewed by Stevens and Maier (2016).

In our context, the main merit of this rapid methodology for screening polyphenols antibacterial activity was to identify polyphenols which, based on their *in vitro* antibacterial activity against foodborne pathogenic or food-spoiling bacterial species, are promising for the preservation of perishable foods.

The difference of susceptibility to polyphenols between Gram-positive and Gram-negative bacteria is a controversial issue. On one hand, many authors like Inouye et al. (2001) concluded that the antibacterial effect of polyphenols was generally more effective against Gram-positive bacteria than Gram negative ones. They indicated that Gram-negative bacteria are more resistant to plant secondary metabolites including phenolics due to the cell wall they possess linked to an outer complex membrane, which slows down the passage of chemicals (Inouye et al., 2001). Nevertheless, this outer membrane may also be altered by some polyphenols (Helander et al., 1998; Lambert et al., 2001; La Storia et al., 2011). On the other hand, Taguri et al. (2006) did not observe any clear correlation between Gram-staining and bacterial susceptibility to polyphenols. In the present study, a 1 g L^−1^ polyphenol concentration resulted in a BLD exceeding 50% for 48.5 and 31.4% of antibacterial activity assays with Gram-positive and Gram-negative bacteria, respectively. However, this difference was not statistically significant since we only considered 3 Gram-negative and 3 Gram-positive species. Indeed, as underlined in the results section and consistently with Taguri et al. (2006) observations, the susceptibility was mainly dependent on the species of bacteria. Based on the proportion of assays resulting in a BLD exceeding 50% (stated between brackets), the 6 bacterial species can be ranked by decreasing susceptibility to the 35 polyphenols: *L. monocytogenes* (57.1%) > *B. subtilis, S*. Enteritidis, and *S. aureus* (45.7% for these 3 bacterial strains) > *E. coli* (31.4%) > *P. aeruginosa* (17.1%).

The lower susceptibility to many polyphenols of *P. aeruginosa* compared to other bacterial strains likely results not only from the outer membrane impermeability of this Gram-negative species but also from a synergy of this limited permeability to polyphenols with chromosomally-encoded multidrug efflux pumps as reviewed by Poole (2001).

Taken together, our results reflected the complexity of the phenomena involved in the antimicrobial activity. The sensitivity of bacteria to antimicrobial agents is known to be dose-dependent but also to be strain-dependent (Rawat et al., 2016; Dantas Silva et al., 2017; Ouerghemmi et al., 2017).

### QSAR Modeling and Bacterial Cells Surface Properties

Despite the heterogeneity of the molecular structure of the set of 35 polyphenols which were considered (3 stilbenes, 8 cinnamic and 6 benzoic acids, 11 flavonoids, 5 coumarins, and 2 naphtoquinones) and consequently the variability of the physico-chemical properties of polyphenols, reliable QSAR models were developed for 4 out of the 6 bacterial strains. Although the models differed from one bacterial strain to another one, namely for the 2 Gram-positive bacterial strains the 6 physico-chemical parameters used in each model comprised hydrophobicity, electric and electronic parameters. Increase of hydrophobicity was associated with an increase of bacterial growth inhibition percentage (BLD) for the 4 bacterial strains. The parameters associated with hydrophobicity were “log D at pH 5.5″ for both Gram-positive strains and “−0.05 < S < 0” for both Gram-negative strains. Despite the diversity of the mechanisms of action of the 35 polyphenols, the fact that reliable models based on a limited number of independent physico-chemical descriptors could be obtained substantiates the hypothesis that the dominant mechanism of action against bacteria for most of polyphenols would be based on their accumulation on their surface which would be favored by their hydrophobicity.

In order to get some insight regarding the characteristics of the surface of the cells of the 4 bacterial species for which reliable QSAR models were obtained, their respective affinity for hexane, hexadecane, chloroform and ethyl acetate were determined. Although this study is very preliminary and other parameters such as the zeta-potential of bacterial cells of the 4 species should also be determined and a larger number of bacterial strains should be investigated, it is noteworthy that the 2 Gram-negative bacterial strains which had a similar affinity for the 4 solvents, *E. coli* ATCC25922, and *S*. Enteritidis E0220, had similar QSAR models, while the 2 Gram-positive bacteria which had a significantly different affinity for these 4 solvents had also different QSAR models. This observation would be consistent with the hypothesis of the accumulation of polyphenols on the surface of bacterial cells which are susceptible to polyphenols. For instance, Nakayama et al. (2015) proposed that the lower susceptibility to epigallocatechin gallate of some lactic acid bacteria strains compared to other Gram-positive bacteria would namely result from their lower surface hydrophobicity which was correlated with the production of great amounts of exopolysaccharides.

## Conclusion

Polyphenols exhibited very different antibacterial activity against the six microbial strains studied that are representative of the foodborne pathogenic and food spoilage bacteria. The same polyphenol may be effective on one type of Gram-positive (or Gram-negative) strain and ineffective on the other ones indicating strain-dependent effect. This is the case for example of 5,7-dihydroxy-4-phenylcoumarin (HUQ) which exhibited 93.5% BLD against *L. monocytogenes*, 89.9% BLD against *S. aureu*s and a slight bacterial growth-promoting effect (BLD of about −26.1%) for *E. coli*. Moreover, the antibacterial effect could not be clearly related to a class of polyphenols. Generally, *L. monocytogenes* was sensitive to polyphenols whereas *P. aeruginosa* was not. 5,8-dihydroxy-1,4-naphthoquinone and butyl gallate appeared the most effective polyphenols exhibiting high antibacterial effect among at least five among the six bacterial strains tested.

The chemometric exploitation of this large dataset of 35 polyphenols allowed developing QSAR models suitable for the prediction of the BLD for *E. coli, S*. Enteritidis, *S. aureu*s, and *B. subtilis*. Satisfactory models were obtained with respect to the variability of the measured BLD and regardless of polyphenol class or the mechanism of toxic action involved. The main descriptors included in the QSAR models were the lipophilicity and the electronic and charge properties of the polyphenols. The models developed for the two Gram-negative bacteria were comparable suggesting similar mechanisms of toxic action while no clear connection was made between Gram-positive ones. Interestingly, strains for which important differences regarding the descriptors included in the QSAR models were observed had also different microbial adhesions to the 4 solvents which were considered. However, more in-depth studies regarding the surface properties of these bacteria and a study with a larger number of bacterial strains with different surface properties should be performed in order to further explore the relationship between QSAR models descriptors and the physico-chemical properties of the surface of bacterial cells.

## Author Contributions

CB and PL conceived and designed the work and drafted the manuscript. NO and PD revised the work critically for important intellectual content. LB-C and LL-A performed antibacterial activity assays as well as microbial adhesion to solvents experiments. VF and YC calculated all physico-chemical descriptors of polyphenols. CB and PL analyzed and interpreted the results. LB-C, NO, and PD revised the manuscript. All authors read and approved the final manuscript.

### Conflict of Interest Statement

The authors declare that the research was conducted in the absence of any commercial or financial relationships that could be construed as a potential conflict of interest.

## References

[B1] Andrade M. Benfeito S. Soares P. Magalhãese Silva D. Loureiro J. Borges A. (2015). Fine-tuning of the hydrophobicity of caffeic acid: studies on the antimicrobial activity against *Staphylococcus aureus* and *Escherichia coli*. RSC Adv. 5, 53915–53925. 10.1039/C5RA05840F

[B2] Bellon-Fontaine M.-.N. Rault J. van Oss C. J. (1996). Microbial adhesion to solvents: a novel method to determine the electron-donor/electron acceptor or Lewis acid-base properties of microbial cells. Coll. Surf. B: Biointerf. 7, 47–53. 10.1016/0927-7765(96)01272-6

[B3] Beltrame P. Beltrame P. L. Carniti P. Guardione D. Lanzetta C. (1988). Inhibiting action of chlorophenols on biodegradation of phenol and its correlation with structural properties of inhibitors. Biotechnol. Bioeng. 31, 821–828. 10.1002/bit.260310809 18584686

[B4] Borges A. Ferreira C. Saavedra M. J. (2013). Antibacterial activity and mode of action of ferulic and gallic acids against pathogenic bacteria. Microb. Drug Resist. 19, 256–265. 10.1089/mdr.2012.0244 23480526

[B5] Bouarab-Chibane L. Degraeve P. Ferhout H. Bouajila J. Oulahal N. (2019). Plant antimicrobial polyphenols as potential natural food preservatives. J. Sci. Food Agric. 99, 1457-1474. 10.1002/jsfa.9357 30206947

[B6] Chirico N. Gramatica P. (2011). Real external predictivity of QSAR models: how to evaluate it? Comparison of different validation criteria and proposal of using the concordance correlation coefficient. J. Chem. Inf. Model. 51, 2320–2335. 10.1021/ci200211n 21800825

[B7] Chirico N. Gramatica P. (2012). Real external predictivity of QSAR models. Part 2. New intercomparable thresholds for different validation criteria and the need for scatter plot inspection. J. Chem. Inf. Model. 52, 2044–2058. 10.1021/ci300084j 22721530

[B8] Chung K. T. Lu Z. Chou M. W. (1998). Mechanism of inhibition of tannic acid and related compounds on the growth of intestinal bacteria. Food Chem. Toxicol. 36, 1053–1060. 10.1016/S0278-6915(98)00086-6 9862646

[B9] Cushnie T. T. Lamb A. J. (2011). Recent advances in understanding the antibacterial properties of flavonoids. Int. J. Antimicrob. Agents 38, 99–107. 10.1016/j.ijantimicag.2011.02.014 21514796

[B10] Daglia M. (2012). Polyphenols as antimicrobial agents. Curr. Opin. Biotechnol. 23, 174–181. 10.1016/j.copbio.2011.08.007 21925860

[B11] Dantas Silva R. P. Machado B. A. Barreto G. A. Costa S. S. Andrade L. N. Amaral R. G.. (2017). Antioxidant, antimicrobial, antiparasitic and cytotoxic properties of various Brazilian propolis extracts. PLoS ONE 12:e0172585. 10.1371/journal.pone.0172585 28358806 PMC5373518

[B12] Djilani A. Dicko A. (2012). The therapeutic benefits of essential oils, in Nutrition, well-being and health in Technology, ed Jaouad (Rijeka: InTech), 155–178.

[B13] Dorman H. J. Deans S. G. (2000). Antimicrobial agents from plants: antibacterial activity of plant volatile oils. J. Appl. Microbiol. 88, 308–316. 10.1046/j.1365-2672.2000.00969.x 10736000

[B14] Duggirala S. Nankar R. P. Rajendran S. Doble M. (2014). Phytochemicals as inhibitors of bacterial cell division protein FtsZ: coumarins are promising candidates. Appl. Biochem. Biotechnol. 174, 283–296. 10.1007/s12010-014-1056-2 25062781

[B15] Engels C. Schieber A. Ganzle M. G. (2012). Sinapic acid derivatives in defatted oriental mustard (*Brassica juncea* L.) seed meal extracts using UHPLC-DAD-ESIMS and identification of compounds with antibacterial activity. Eur. Food Res. Technol. 234, 535–542. 10.1007/s00217-012-1669-z

[B16] Fang Y. Lu Y. Zang X. Wu T. Qi X. Pan S.. (2016). 3D-QSAR and docking studies of flavonoids as potent *Escherichia coli* inhibitors. Sci. Rep. 6:23634. 10.1038/srep23634 27049530 PMC4822154

[B17] Griffin S. G. Wyllie S. G. Markham J. L. (2005). Antimicrobially active terpenes cause K^+^ leakage in *E. coli* cells. J. Essent. Oil. Res. 17, 686–690. 10.1080/10412905.2005.9699033

[B18] Gyawali R. Ibrahim S. A. (2014). Impact of plant derivatives on the growth of foodborne pathogens and the functionality of probiotics. Appl. Microbiol. Biotechnol. 95, 29–45. 10.1007/s00253-012-4117-x 22622837

[B19] Haraguchi H. Tanimoto K. Tamura Y. Mizutani K. Kinoshita T. (1998). Mode of antibacterial action of retrochalcones from *Glycyrrhiza inflata*. Phytochem. 48, 125–129. 10.1016/S0031-9422(97)01105-9 9621457

[B20] Helander I. M. Alakomi H. Latva-Kala K. Mattila-Sandholm T. Pol I. Smid E. J. (1998). Characterization of the action of selected essential oil components on Gram-negative bacteria. J. Agric. Food Chem. 46, 3590–3595. 10.1021/jf980154m

[B21] Ikigai H. Nakae T. Hara Y. Shimamura T. (1993). Bactericidal catechins damage the lipid bilayer. Biochim. Biophys. Acta 1147, 132–136. 10.1016/0005-2736(93)90323-R 8466924

[B22] Inouye S. Yamaguchi H. Takizawa T. (2001). Screening of the antibacterial effects of variety of essential oils on respiratory tract pathogens using a modified dilution assay method. J. Infect. Chemother. 7, 251–254. 10.1007/s101560170022 11810593

[B23] Kim J.-K. Kim N. Lim Y. H. (2010). Evaluation of the antibacterial activity of rhapontigenin produced from rhapontin by biotransformation against *Propionibacterium acnes*. J. Microbiol. Biotechnol. 20, 82–87. 10.4014/jmb.0907.07022 20134237

[B24] Kocevar Glavac N. Lunder M. (2018). Preservative efficacy of selected antimicrobials of natural origin in a cosmetic emulsion. Int. J. Cosmetic Sci. 40, 276–284. 10.1111/ics.12461 29729020

[B25] Kubinyi H. (1994). Variable selection in QSAR Studies. I. An evolutionary algorithm. Quant. Struct. Act. Relat. 13, 285–294. 10.1002/qsar.19940130306

[B26] Kuete V. Alibert-Franco S. Eyong K. O. (2011). Antibacterial activity against bacteria expressing a multidrug-resistant phenotype. Int. J. Antimicrob. Agents. 37, 156–161. 10.1016/j.ijantimicag.2010.10.020 21163632

[B27] Kurita N. Miyaji M. Kurane R. Takahara Y. (1981). Antifungal activity of components of essential oils. Agric. Biol. Chem. 45, 945–952.

[B28] La Storia A. Ercolini D. Marinello F. Di Pasqua R. Villani F. Mauriello G. (2011). Atomic force microscopy analysis shows surface structure changes in carvacrol-treated bacterial cells. Res. Microbiol. 162, 164–172. 10.1016/j.resmic.2010.11.006 21168481

[B29] Lambert R. J. Skandamis P. N. Coote P. J. Nychas G. J. (2001). A study of the minimum inhibitory concentration and mode of action of oregano essential oil, thymol and carvacrol. J. Appl. Microbiol. 91, 453–462. 10.1046/j.1365-2672.2001.01428.x 11556910

[B30] Larif M. Ouhssine M. Soulaymani A. Elmidaoui A. (2015). Potential effluent oil mills and antibacterial activity polyphenols against some pathogenic strains. Res. Chem. Intermed. 41, 1213–1225. 10.1007/s11164-013-1267-0

[B31] Lee B. H. Hébraud M. Bernardi T. (2017). Increased adhesion of *Listeria monocytogenes* strains to abiotic surfaces under cold stress. Front. Microbiol 8:2221. 10.3389/fmicb.2017.02221 29187836 PMC5695204

[B32] Levet A. Bordes C. Clément Y. Mignon P. Chermette H. Marote P.. (2013). Quantitative structure-activity relationship to predict acute fish toxicity of organic solvents. Chemosphere 93, 1094–1103. 10.1016/j.chemosphere.2013.06.002 23866172

[B33] Levet A. Bordes C. Clément Y. Mignon P. Morell C. Chermette H.. (2016). Acute aquatic toxicity of organic solvents modeled by QSARs. J. Mol. Model. 22:288. 10.1007/s00894-016-3156-0 27830479

[B34] Li A. N. Li S. Zhang Y. J. Xu X. R. Chen Y. M. Li H. B. (2014). Resources and biological activities of natural polyphenols. Nutrients. 6, 6020–6047. 10.3390/nu6126020 25533011 PMC4277013

[B35] Lumbiny B. J. Hui Z. Islam M. A. (2014). Antiaging, antioxidant flavonoids; synthesis, antimicrobial screening as well as 3D QSAR CoMFA models for the prediction of biological activity. J. Asiat. Soc. Bangladesh Sci. 39, 191–199. 10.3329/jasbs.v39i2.17856

[B36] Medina L. F. Hertz P. F. Stefani V. Henriques J. A. Zanotto-Filho A. Brandelli A. (2006). Aminonaphtoquinone induces oxidative stress in *Staphylococcus aureus*. Biochem. Cell. Biol. 84, 720–727. 10.1139/o06-087 17167535

[B37] Mercader A. G. Duchowicz P. R. Fernandez F. M. Castro E. A. (2008). Modified and enhanced replacement method for the selection of molecular descriptors in QSAR and QSPR theories. Chemom. Intell. Lab. Syst. 92, 138–144. 10.1016/j.chemolab.2008.02.005

[B38] Miceli A. Aleo A. Corona O. Sardina M. T. Mammina C. Settanni L. (2014). Antibacterial activity of *Borago officinalis* and *Brassica juncea* aqueous extracts evaluated *in vitro* and *in situ* using different food model systems. Food Control 40, 157–164. 10.1016/j.foodcont.2013.12.006

[B39] Moleyar V. Narasimham P. (1986). Antifungal activity of some essential oil components. Food Microbiol. 3, 331–336. 10.1016/0740-0020(86)90017-1 1457292

[B40] Nakayama M. Tomiyama D. Shigemune N. Mitani A. Xu W. Miyamoto T. (2015). Cell surface hydrophobicity contributes to lactobacillus tolerance to antibacterial actions of catechins. Food Sci. Technol. Res. 21, 583–588. 10.3136/fstr.21.583

[B41] National Committee for Clinical Laboratory Standards (2004). Performance Standards for Antimicrobial Susceptibility Testing, 14th Informational Supplement. Approved Standard M100eS14. Wayne, PA: NCCLS.

[B42] Negi P. S. (2012). Plant extracts for the control of bacterial growth: Efficacy, stability, and safety issues for food application. Int. J. Food Microbiol. 156, 7–17. 10.1016/j.ijfoodmicro.2012.03.006 22459761

[B43] OECD (2004). OECD Series on Testing and Assessment; Number 49; The Report From the Expert Group on QSARs on the Principles for the Validation of QSARs.

[B44] OECD (2007). OECD Series on Testing and Assessment; Number 69; Guidance Document on the Validation of (Quantitative) Structure-Activity Relationship [(Q) SAR] Models.

[B45] Ouerghemmi I. Bettaieb Rebey I. Rahali F. Z. Bourgou S. Pistelli L. Ksouri R.. (2017). Antioxidant and antimicrobial phenolic compounds from extracts of cultivated and wild-grown Tunisian *Ruta chalepensis*. J. Food. Drug. Anal. 25, 350–359. 10.1016/j.jfda.2016.04.001 28911677 PMC9332523

[B46] Oyedemi S. O. Okoh A. I. Mabinya L. V. Pirochenva G. Afolayan A. J. (2009). The proposed mechanism of bactericidal action of eugenol, (-terpineol and (-terpinene against *Listeria monocytogenes, Streptococcus pyogenes, Proteus vulgaris* and *Escherichia coli*. African J. Biotechnol. 8, 1280–1290. 10.4314/ajb.v8i7.60106

[B47] Pelczar M. J. Chan E. C. S. Krieg N. R. (1988). Control of microorganisms, the control of microorganisms by physical agents, in Microbiology, eds Chan E. C. S. Krieg N. R. (New York, NY: Mc Graw-Hill International), 469–509.

[B48] Perdew J. P. Burke K. Ernzerhof M. (1996). Generalized gradient approximation, made simple. Phys. Rev. Lett. 77, 3865–3868. 10.1103/PhysRevLett.77.3865 10062328

[B49] Plumed-Ferrer C. Väkeväinen K. Komulainen H. Rautiainen M. Smeds A. Raitanen J. E. (2013). The antimicrobial effects of wood-associated polyphenols on food pathogens and spoilage organisms. Int. J. Food Microbiol. 166, 99–107. 10.1016/j.ijfoodmicro.2013.06.031 23624538

[B50] Poole K. (2001). Multidrug efflux pumps and antimicrobial resistance in *Pseudomonas aeruginosa* and related organisms. J. Mol. Microbiol. Biotechnol. 3, 255–264. Available online at: https://www.karger.com/Journal/Home/22839 11321581

[B51] Quévrain E. Domart-Coulon I. Pernice M. Bourguet-Kondracki M. L. (2009). Novel natural parabens produced by a microbulbifer bacterium in its calcareous sponfe host leuconia nivea. Environ. Microbiol. 11, 1527–1539. 10.1111/j.1462-2920.2009.01880.x 19226298

[B52] Radulovic N. S. Blagoje vic P. D. Stojanovic-Radic Z. Z. Stojanovic N. M. (2013). Antimicrobial plant metabolites: structure diversity and mechanism of action. Curr. Med. Chem. 20, 932–952. 10.2174/092986713805219136 23210781

[B53] Rawat S. Jugran A. K. Bahukhandi A. Bahuguna A. Bhatt I. D. Rawal R. S. (2016). Antioxidant and antimicrobial properties of some ethno-therapeutically important medicinal plants of Indian Himalayan Region. 3 Biotech. 6:154 10.1007/s13205-016-0470-2 PMC4949164 28330226

[B54] Roy P. P. Paul S. Mitra I. Roy K. (2009). On-two novel parameters for validation of predictive QSAR models. Molecules 14, 1660–1701. 10.3390/molecules14051660 19471190 PMC6254296

[B55] Schüürmann G. Ebert R. U. Chen J. Wang B. Kühne R. (2008). External validation and prediction employing the predictive squared correlation coefficient - test set activity mean *vs* training set activity mean. J. Chem. Inf. Model. 48, 2140–2145. 10.1021/ci800253u 18954136

[B56] Sierra-Alvarez R. Lettinga G. (1991). The effect of aromatic structure on the inhibition of acetolastic methanogenesis in granular sludge. Appl. Microbiol. Biotechnol. 34, 544–550. 10.1007/BF00180586

[B57] Sikkema J. de Bont J. A. Poolman B. (1995). Mechanisms of membrane toxicity of hydrocarbons. Microbiol. Rev. 59, 201–222. 7603409 10.1128/mr.59.2.201-222.1995 PMC239360

[B58] Stapleton P. D. Shah S. Hamilton-Miller J. M. T. (2004). Anti-*Staphylococcus aureus* activity and oxacillin resistance modulating capacity of 3- O-acylcatechins. Int. J. Antimicrob. Agents 24, 374–380. 10.1016/j.ijantimicag.2004.03.024 15380264

[B59] Stevens J. F. Maier C. S. (2016). The chemistry of gut microbial metabolism of polyphenols. Phytochem. Rev. 15, 425–444. 10.1007/s11101-016-9459-z 27274718 PMC4888912

[B60] Taguri T. Tanaka T. Kouno I. (2006). Antibacterial spectrum of plant polyphenols and extracts depending upon hydroxyphenyl structure. Biol. Pharm. Bull. 29, 2226–2235. 10.1248/bpb.29.2226 17077519

[B61] Tsuchiya H. Sato M. Miyazaki T. Fujiwara S. Tanigaki S. Ohyama M. (1996). Comparative study on the antibacterial activity of phytochemical flavanones against methicillin-resistant *Staphylococcus aureus*. J. Ethnopharmacol. 50, 27–34. 10.1016/0378-8741(96)85514-0 8778504

[B62] Upadhyay A. Upadhyaya I. Kollanoor-Johny A. Venkitanarayanan K. (2014). Combating pathogenic microorganisms using plant-derived antimicrobials: a minireview of the mechanistic basis. BioMed. Res. Int. 2014:761741. 10.1155/2014/761741 25298964 PMC4178913

[B63] Ya C. Gaffney S. H. Lilley T. H. Haslam E. (1988). Carbohydrate-polyphenol complexation, in Chemistry and Significance of Condensed Tannins, eds Hemingway R. W. Karchesy J. J. (New York, NY: Plenum Press), 553.

[B64] Yoda Y. Hu Z. Q. Zhao W. H. Shimamura T. (2004). Different susceptibilities of *Staphylococcus* and gram-negative rods to epigallocatechin gallate. J. Infect. Chemother. 10, 55–58. 10.1007/s10156-003-0284-0 14991521

